# CHD7 regulates bone-fat balance by suppressing PPAR-γ signaling

**DOI:** 10.1038/s41467-022-29633-6

**Published:** 2022-04-13

**Authors:** Caojie Liu, Qiuchan Xiong, Qiwen Li, Weimin Lin, Shuang Jiang, Danting Zhang, Yuan Wang, Xiaobo Duan, Ping Gong, Ning Kang

**Affiliations:** State Key Laboratory of Oral Diseases, National Clinical Research Center for Oral Diseases, West China Hospital of Stomatology, Sichuan University, Chengdu, 610041 PR China

**Keywords:** Stem-cell differentiation, Diseases, Mesenchymal stem cells

## Abstract

Chromodomain helicase DNA-binding protein 7 (CHD7), an ATP-dependent eukaryotic chromatin remodeling enzyme, is essential for the development of organs. The mutation of *CHD7* is the main cause of CHARGE syndrome, but its function and mechanism in skeletal system remain unclear. Here, we show conditional knockout of *Chd7* in bone marrow mesenchymal stem cells (MSCs) and preosteoblasts leads to a pathological phenotype manifested as low bone mass and severely high marrow adiposity. Mechanistically, we identify enhancement of the peroxisome proliferator-activated receptor (PPAR) signaling in *Chd7*-deficient MSCs. Loss of *Chd7* reduces the restriction of PPAR-γ and then PPAR-γ associates with trimethylated histone H3 at lysine 4 (H3K4me3), which subsequently activates the transcription of downstream adipogenic genes and disrupts the balance between osteogenic and adipogenic differentiation. Our data illustrate the pathological manifestations of *Chd7* mutation in MSCs and reveal an epigenetic mechanism in skeletal health and diseases.

## Abstract (web summary)

CHD7 is chromatin remodeler and mutations of CHD7 are the main cause of CHARGE syndrome. Here the authors show that conditional knockout of Chd7 in bone marrow mesenchymal stem cells (MSCs) and pre-osteoblasts leads to a skeletal system development disorder in mice and upregulated PPAR signaling, disrupting the balance between osteogenic and adipogenic differentiation.

## Introduction

As the common progenitors for osteoblasts and adipocytes, bone marrow mesenchymal stem cells (MSCs) show strict control of cell fate decisions to maintain the balance between osteogenic and adipogenic differentiation to ensure the health of the skeletal system^1,2^. Characterized by low bone mass and extensive marrow adipose tissue (MAT) accumulation, senile osteoporosis in elderly patients leads to a series of complications, e.g., increasing bone fragility and susceptibility to fracture^3–5^. Bone marrow MSCs preferentially differentiate into adipocytes under senility or pathological stimuli, e.g., hormone disorders, leading to increased MAT and progressive bone loss^1,3,6^. Unfortunately, the precise mechanism by which MSC lineage allocation favors adipogenic differentiation toward osteogenic differentiation remains to be explored.

As typical ATP-dependent eukaryotic chromatin remodeling enzymes, the members of the chromodomain helicase DNA-binding protein (CHD) superfamily interrupt the interaction between DNA and chromatin by translocating nucleosomes along the DNA strand^7,8^. The CHD family is essential for the expression of normal genes and the maintenance of dynamic chromatin structure via their ability to activate or suppress specific histone markers^9,10^. Hence, the CHD superfamily is critical for the maintenance and proliferation of stem cells, in addition to cell differentiation and cell fate decisions^11,12^.

As one of the most researched members of the CHD family, CHD7 is widely expressed in various tissues and plays a critical role in the development of organs^10,12^. Mutation of *CHD7* is the main cause of CHARGE syndrome, a kind of developmental disorder involving multiple organ system defects, characterized as coloboma of the eye, heart defects, atresia of the choanae, retarded growth, genital anomalies, ear malformations and deafness^13–15^. The acronym of these six major manifestations was defined as CHARGE syndrome in 1981^16^.

As previously indicated, CHD7 is an ATP-dependent chromatin remodeling enzyme that regulates the location of nucleosomes and alters DNA accessibility^17,18^. According to previous studies, one of the main functions of the CHD family is binding to methylated histone residues and promoting methylated histone H3 at lysine 4 (H3K4me) interactions^8,19^. Previously found by chromatin immunoprecipitation (ChIP), CHD7 recruitment was strongly linked to histone modification, e.g., colocalization with H3K4me1 at the enhancer subregion and with H3K4me3 at the promoters of the transcription initiation site^20^.

We previously showed that CHD7 could promote the osteogenic differentiation of human bone MSCs by interacting with SMAD1 and binding to the enhancer region of *SP7*^21^. In this work, we generated *Chd7* conditional knockout mice and unveiled the essential role of CHD7 in MSC fate decisions by suppressing the peroxisome proliferator-activated receptor gamma (PPAR-γ) signaling pathway. Such findings might provide insight into the pivotal regulatory role of CHD7 in bone development and diseases, as well as a probable clinical therapeutic strategy.

## Results

### Deletion of *Chd7* in MSCs leads to skeletal development disorder

To investigate the potential role of CHD7 in skeletal development, we examined the expression of CHD7 in mouse femurs. Immunohistochemical (IHC) staining showed that CHD7 was prevalently expressed in the nuclei of bone cells and bone marrow in both primary and secondary ossification centers but marginally expressed in chondrocytes of the growth plate (Supplementary Fig. 1a). Immunofluorescence (IF) staining images showed the same trend (Supplementary Fig. 1b).

**Fig. 1:**
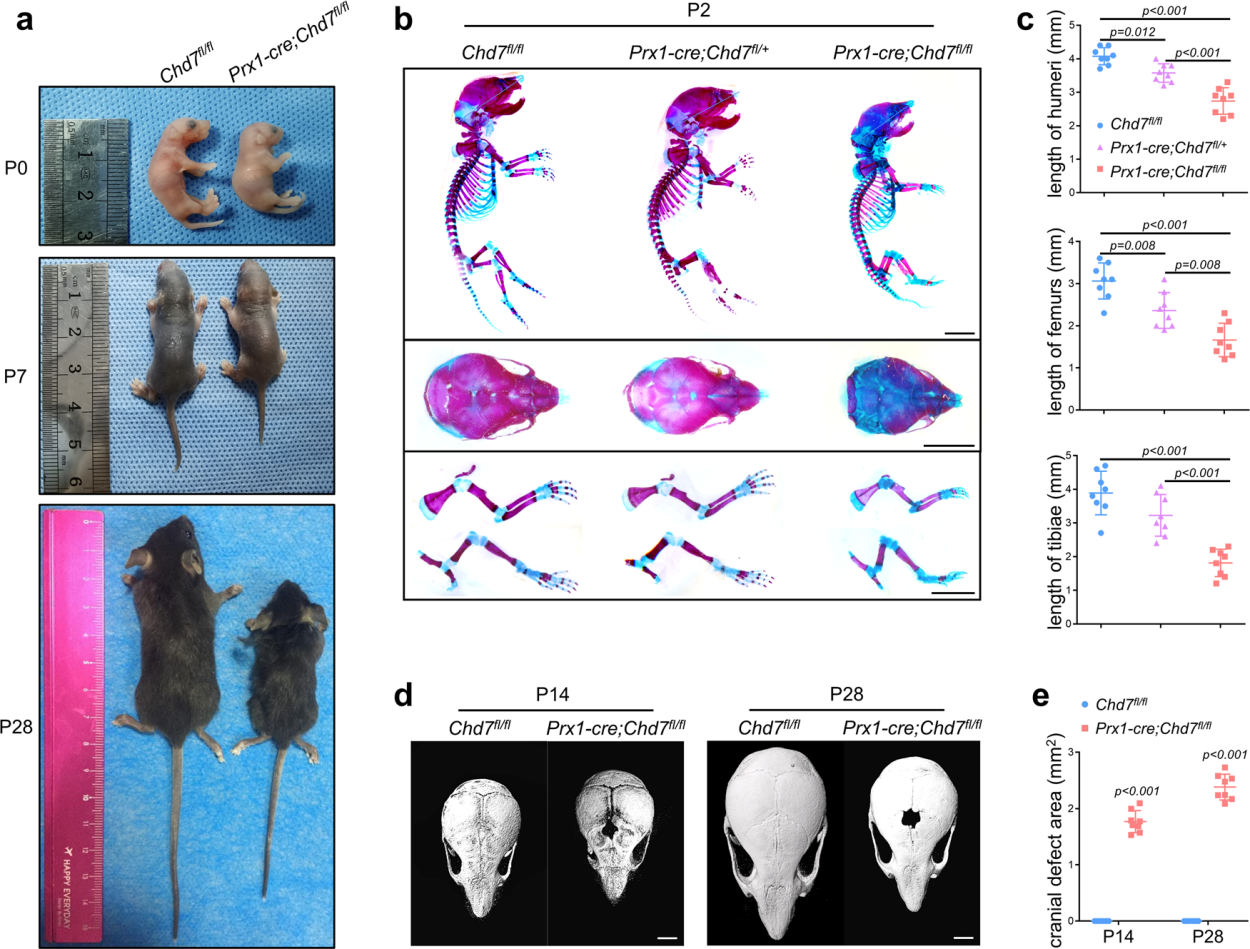
Deletion of *Chd7* in MSCs leads to skeletal development disorder. **a** Representative images of the *Chd7*^*fl/fl*^ and *Prx1-cre;Chd7*^*fl/fl*^ mice at postnatal day 0, day 7, and day 28. **b** Alizarin red/Alcian blue whole-mount skeletal staining images of the *Chd7*^*fl/fl*^, *Prx1-cre;Chd7*^*fl/+*^, and *Prx1-cre;Chd7*^*fl/fl*^ pups at postnatal day 2. Compared to the control littermates, The *Prx1-cre;Chd7*^*fl/fl*^ pups had shorter forelimbs and hindlimbs and a lower degree of mineralization in the cranial and maxillofacial bone. Scale bar, 5 mm. **c** Quantification of (**b**), including the length of humeri, femurs, and tibiae of the mice at postnatal day 2 (*n* = 8). **d** Representative reconstructed microCT images of skulls of the 2-week-old and 4-week-old *Chd7*^*fl/fl*^ and *Prx1-cre;Chd7*^*fl/fl*^ mice. The skulls of *Prx1-cre;Chd7*^*fl/fl*^ mice showed nonclosure of fontanels. Scale bar, 2 mm. **e** Quantification of (**d**), cranial defect area calculated by ImageJ (*n* = 8). Data are shown as the mean ± S.D.; *p* value by two-tailed for independent sample tests or one-way ANOVA with Tukey’s post hoc tests for multiple comparisons.

Next, we generated *Chd7* flox (*Chd7*^*fl/+*^) mice via the CRISPR/Cas9 technique and then mated them with *Prx1-cre* transgenic mice to generate conditional homozygous *Chd7* knockout mice, *Prx1-cre;Chd7*^*fl/fl*^^3,22–27^ (Supplementary Fig. 1c, d). The birth status and the gender ratio of the pups were basically consistent with Mendelian law. *Prx1-cre;Chd7*^*fl/fl*^ mice were viable, although with a relatively lower survival rate (Supplementary Fig. 1e). The *Prx1-cre;Chd7*^*fl/fl*^ mice had a smaller size, a lighter weight, and delayed growth features compared to their *Chd7*^*fl/fl*^ control littermates at 4 weeks after birth (Fig. 1a, Supplementary Fig. 1f). The IHC and IF staining images of the *Prx1-cre;Chd7*^*fl/fl*^ mice confirmed the successful deletion of *Chd7* in MSCs (Supplementary Fig. 1a, b).

We conducted whole-mount skeletal staining on pups 2 days after birth and observed that the skeletal development of *Prx1-cre;Chd7*^*fl/fl*^ pups is significantly delayed compared with that of *Chd7*^*fl/fl*^ littermates, with shorter forelimbs and hindlimbs and a lower degree of mineralization in the cranial and maxillofacial bone (Fig. 1b, c). The skulls of 2-week-old and 4-week-old mice were harvested and scanned by microcomputed tomography (microCT). The reconstructed images showed nonclosure of fontanels in the *Prx1-cre;Chd7*^*fl/fl*^ mice, indicating that knockout of *Chd7* in MSCs leads to cranial bone hypoplasia (Fig. 1d, e).

### Deletion of *Chd7* in MSCs leads to low bone mass and high marrow adiposity

**Fig. 2:**
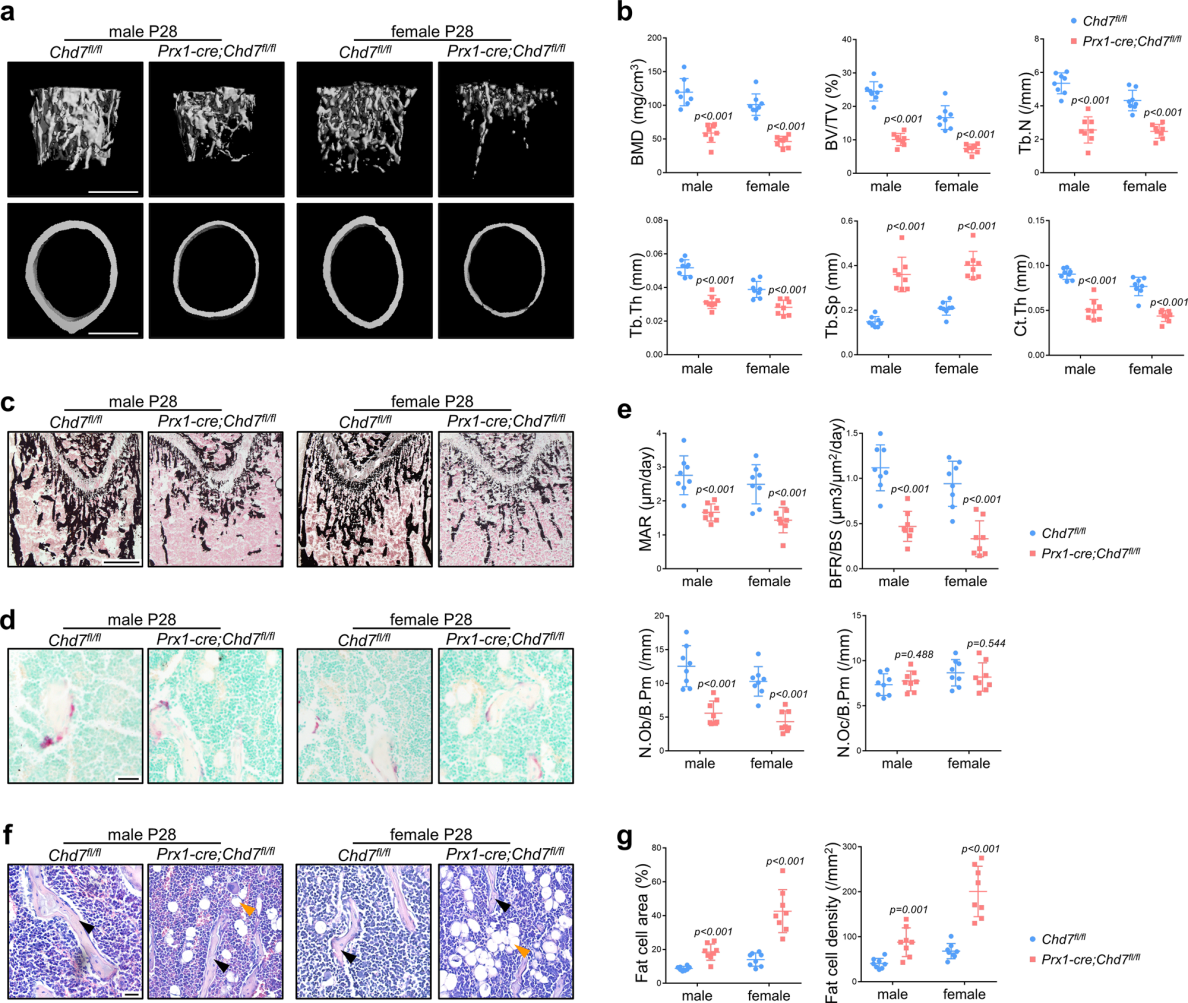
Deletion of *Chd7* in MSCs leads to low bone mass and high marrow adiposity. **a** Representative microCT images of distal femurs and midshaft cortical bone from *Chd7*^*fl/fl*^ and *Prx1-cre;Chd7*^*fl/fl*^ mice at 4 weeks old (Scale bar, 500 μm). **b** Quantitative microCT analyses of the distal end of the femurs from *Chd7*^*fl/fl*^ and *Prx1-cre;Chd7*^*fl/fl*^ mice at 4 weeks old (*n* = 8). **c** Von Kossa staining of undecalcified sections of femurs from *Chd7*^*fl/fl*^ and *Prx1-cre;Chd7*^*fl/fl*^ mice at 4 weeks old (Scale bar, 500 μm). **d** TRAP staining of femur sections from *Chd7*^*fl/fl*^ and *Prx1-cre;Chd7*^*fl/fl*^ mice at 4 weeks old (Scale bar, 50 μm). **e** Dynamic osteogenic index of trabecular bone from the femoral metaphysis in *Chd7*^*fl/fl*^ and *Prx1-cre;Chd7*^*fl/fl*^ mice at 4 weeks old, including MAR and BFR determined by double labeling (*n* = 8). Histomorphological analysis of trabecular bone from the femoral metaphysis in *Chd7*^*fl/fl*^ and *Prx1-cre;Chd7*^*fl/fl*^ mice at 4 weeks old, including osteoblast and osteoclast numbers (*n* = 8). **f** Representative images of adipocytes in the distal femur marrow in *Chd7*^*fl/fl*^ and *Prx1-cre;Chd7*^*fl/fl*^ mice at 4 weeks old. Blacks arrows indicate trabecular bones, and orange arrows indicate marrow adipose tissues (Scale bar, 50 μm). **g** Quantitative measurements of adipocytes, including number and area of adipocytes in the distal marrow per tissue area. Quantitative data were obtained using the ImageJ software (*n* = 8). Data are shown as the mean ± S.D.; *p* value by two-tailed Student’s *t* test.

MicroCT analysis of distal femur metaphysis indicated that bone mineral density (BMD) and bone volume/tissue volume ratio (BV/TV) were significantly reduced in both male and female *Prx1-cre;Chd7*^*fl/fl*^ mice compared to their *Chd7*^*fl/fl*^ littermates at 4 weeks of age. In addition, *Chd7* deletion impaired the trabecular number (Tb.N), the trabecular thickness (Tb.Th) and the midshaft cortical thickness (Ct.Th), while elevating the trabecular separation (Tb.Sp) (Fig. 2a, b).

The undecalcified sections after Von Kossa staining further validated the low bone mass phenotype of the *Prx1-cre;Chd7*^*fl/fl*^ mice (Fig. 2c). The *Prx1-cre;Chd7*^*fl/fl*^ mice also showed a slower mineral apposition rate (MAR) and bone formation rate (BFR) (Fig. 2e). In addition, the *Prx1-cre;Chd7*^*fl/fl*^ mice had fewer osteoblasts (N.Ob/B.Pm), indicating a diminished bone formation. The osteoclast number (N.Oc/B.Pm) showed no obvious differences between groups (Fig. 2d, e). *Chd7* knockout also led to a slightly shortened growth plate (Supplementary Fig. 1a).

Notably, in both male and female *Prx1-cre;Chd7*^*fl/fl*^ mice, the loss of bone mass was associated with obviously increased bone marrow adipose tissue accumulation in both the primary and secondary ossification centers (Fig. 2f). The number and density of bone marrow adipocytes were apparently elevated in the *Prx1-cre;Chd7*^*fl/fl*^ mice (Fig. 2g). For unknown reasons, the phenotype of increased MAT was more striking in female *Prx1-cre;Chd7*^*fl/fl*^ mice.

### Loss of *Chd7* in preosteoblasts leads to low bone mass and high marrow adiposity

**Fig. 3:**
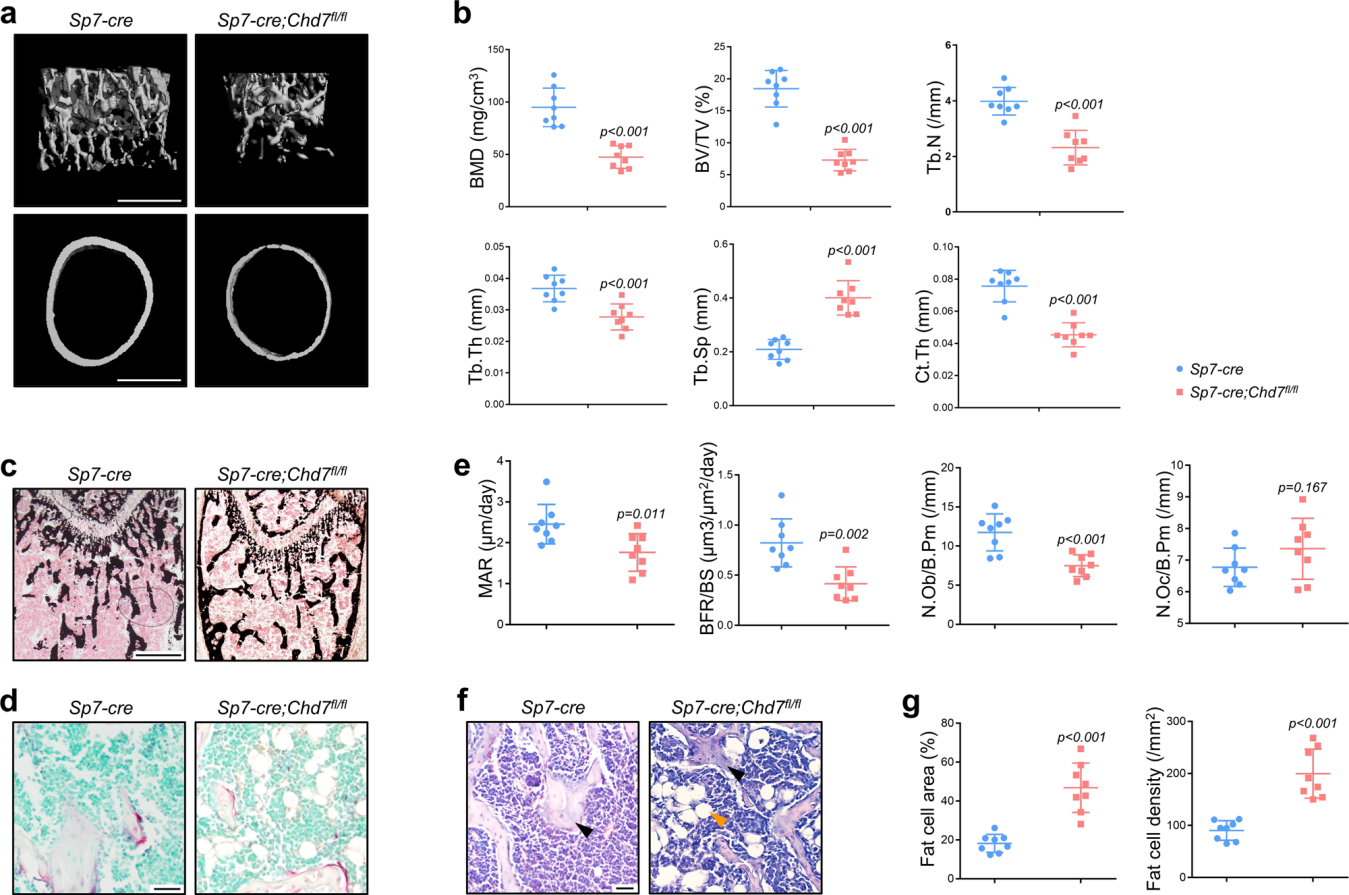
Loss of *Chd7* in preosteoblasts leads to low bone mass and high marrow adiposity. **a** Representative microCT images of distal femurs and midshaft cortical bone from *Sp7-cre* and *Sp7-cre;Chd7*^*fl/fl*^ mice at 4 weeks old (Scale bar, 500 μm). **b** Quantitative microCT analyses of the distal end of the femurs from *Sp7-cre* and *Sp7-cre;Chd7*^*fl/fl*^ mice at 4 weeks old (*n* = 8). **c** Von Kossa staining of undecalcified sections of femurs from *Sp7-cre* and *Sp7-cre;Chd7*^*fl/fl*^ mice at 4 weeks old (Scale bar, 500 μm). **d** TRAP staining of femur sections from *Sp7-cre* and *Sp7-cre;Chd7*^*fl/fl*^ mice at 4 weeks old (Scale bar, 50 μm). **e** Dynamic osteogenic index of trabecular bone from the femoral metaphysis in *Sp7-cre* and *Prx1-cre;Chd7*^*fl/fl*^ mice at 4 weeks old, including MAR and BFR determined by double labeling (*n* = 8). Histomorphological analysis of trabecular bone from the femoral metaphysis in *Sp7-cre* and *Prx1-cre;Chd7*^*fl/fl*^ mice at 4 weeks old, including osteoblast and osteoclast numbers (*n* = 8). **f** Representative images of adipocytes in the distal femur marrow in *Sp7-cre* and *Prx1-cre;Chd7*^*fl/fl*^ mice at 4 weeks old. Blacks arrows indicate trabecular bones, and orange arrows indicate marrow adipose tissues (Scale bar, 50 μm). **g** Quantitative measurements of adipocytes, including number and area of adipocytes in the distal marrow per tissue area. Quantitative data were obtained using the ImageJ software (*n* = 8). Data are shown as the mean ± S.D.; *p* value by two-tailed Student’s *t* test.

To further investigate whether depletion of *Chd7* from committed osteoblast progenitors could induce a similar bone loss phenotype, we mated the *Sp7-cre* transgenic mice with the *Chd7*^*fl/fl*^ mice^28^. The *Sp7-cre;Chd7*^*fl/fl*^ mice also exhibited a smaller size, lighter weight, lower survival rate and delayed growth features (Supplementary Fig. 2a, b). MicroCT analysis of the femur metaphysis revealed a diminishment in trabecular and cortical bone in the *Sp7-cre;Chd7*^*fl/fl*^ mice, as shown by impaired skeletal parameters, e.g., BMD, BV/TV, Tb.N, Tb.Th and Ct.Th, and elevated Tb.Sp (Fig. 3a, b).

The Von Kossa staining validated the low bone mass phenotype of the *Sp7-cre;Chd7*^*fl/fl*^ mice (Fig. 3c). The *Sp7-cre;Chd7*^*fl/fl*^ mice also showed declined dynamic skeletal histomorphometric features, manifested as slower MAR and BFR (Fig. 3e). In addition, the decrease in osteoblast numbers further confirmed the osteopenic manifestations of the *Sp7-cre;Chd7*^*fl/fl*^ mice (Fig. 3d, e).

Similarly, increased MAT accumulation was also observed in both the primary and secondary ossification centers of femurs from the *Sp7-cre;Chd7*^*fl/fl*^ mice (Fig. 3f, g). Moreover, the MAT accumulation phenomena were more remarkable in the tibia of the *Sp7-cre;Chd7*^*fl/fl*^ mice at the age of 4 weeks. Considerable MAT was observed at the mesial region, while the distal region was mostly occupied by MAT, with a very small amount of bone tissue left at the distal region of tibiae in the female *Sp7-cre;Chd7*^*fl/fl*^ mice (Supplementary Fig. 2c).

### *Chd7*-deficient MSCs exhibit reduced osteogenic and increased adipogenic potential

Next, we sought to isolate MSCs from the *Chd7*^*fl/fl*^ and *Prx1-cre;Chd7*^*fl/fl*^ mice and compared their osteogenic and adipogenic potential in vitro (Supplementary Fig. 3a). Flow cytometry was conducted to identify the primary mouse bone marrow mesenchymal stem cells with anti-CD29 and anti-CD90.2 antibodies.

**Fig. 4:**
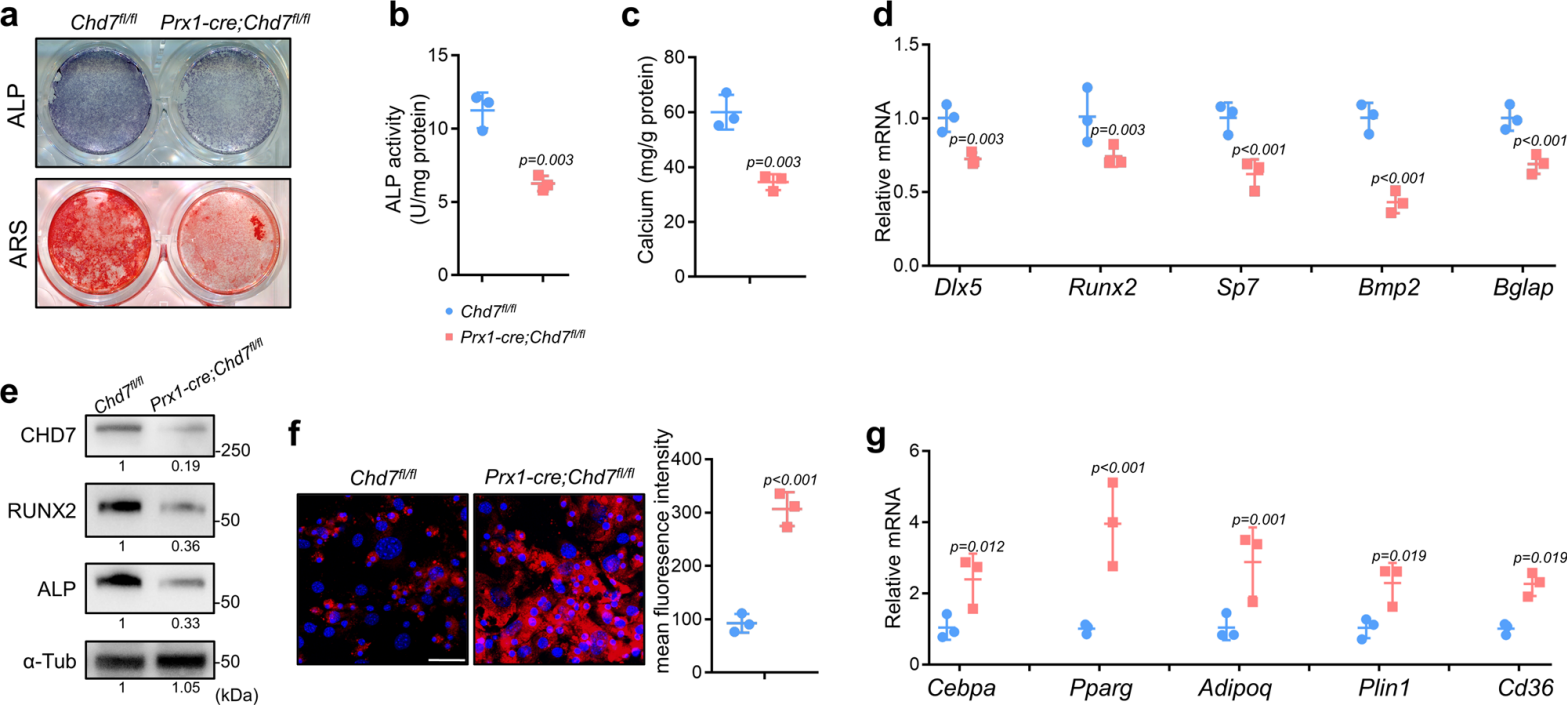
*Chd7*-deficient MSCs exhibit reduced osteogenic and increased adipogenic potential. **a** Representative images of ALP and ARS staining in MSCs. **b** Quantitative analyses of the ALP activity (*n* = 3 biologically independent MSC sample). **c** Quantitative analyses of the mineralization (*n* = 3 biologically independent MSC sample). **d** qRT-PCR analyses of the mRNA expression of *Dlx5, Runx2, Sp7, Bmp2*, and *Bglap* in MSCs under osteogenic conditions (*n* = 3 biologically independent MSC sample) **e** Western blot analysis and quantification of CHD7, RUNX2 and alkaline phosphatase in bone marrow MSCs isolated from the *Chd7*^*fl/fl*^ and *Prx1-cre;Chd7*^*fl/fl*^ mice. **f** Representative images and quantitative analyses of Nile red staining in MSCs. Scale bar, 50 μm (*n* = 3 biologically independent MSC slides). **g** qRT-PCR analyses of the mRNA expression of *Cebpa, Pparg, Adipoq, Plin1, CD36* in MSCs under adipogenic conditions (*n* = 3 biologically independent MSC samples). Data are shown as the mean ± S.D.; *p* value by two-tailed Student’s *t* test.

After osteogenic induction, lighter alkaline phosphatase (ALP) staining indicated the incompetent osteogenic differentiation of the *Chd7*-deleted MSCs (Fig. 4a). Meanwhile, impaired ALP activity and less calcium mineralization were detected in the *Prx1-cre;Chd7*^*fl/fl*^ MSCs (Fig. 4a–c). The significantly downregulated expression of osteogenic markers, e.g., *Dlx5, Runx2, Sp7, Bmp2,* and *Bglap*, strengthened the reduced osteogenic potential of *Chd7*-deficient MSCs (Fig. 4d, e).

After adipogenic induction, the elevated intensity of Nile red staining demonstrated obviously increased lipid droplet in the *Chd7*-deleted MSCs (Fig. 4f). Meanwhile, significantly upregulated expression of classic adipogenic genes, e.g., *Cebpa, Pparg, Adipoq, Plin1*, and *Cd36*, indicated the intensified adipogenic potential of the *Prx1-cre;Chd7*^*fl/fl*^ MSCs (Fig. 4g).

### CHD7 suppresses the transactivation of PPAR-γ

**Fig. 5:**
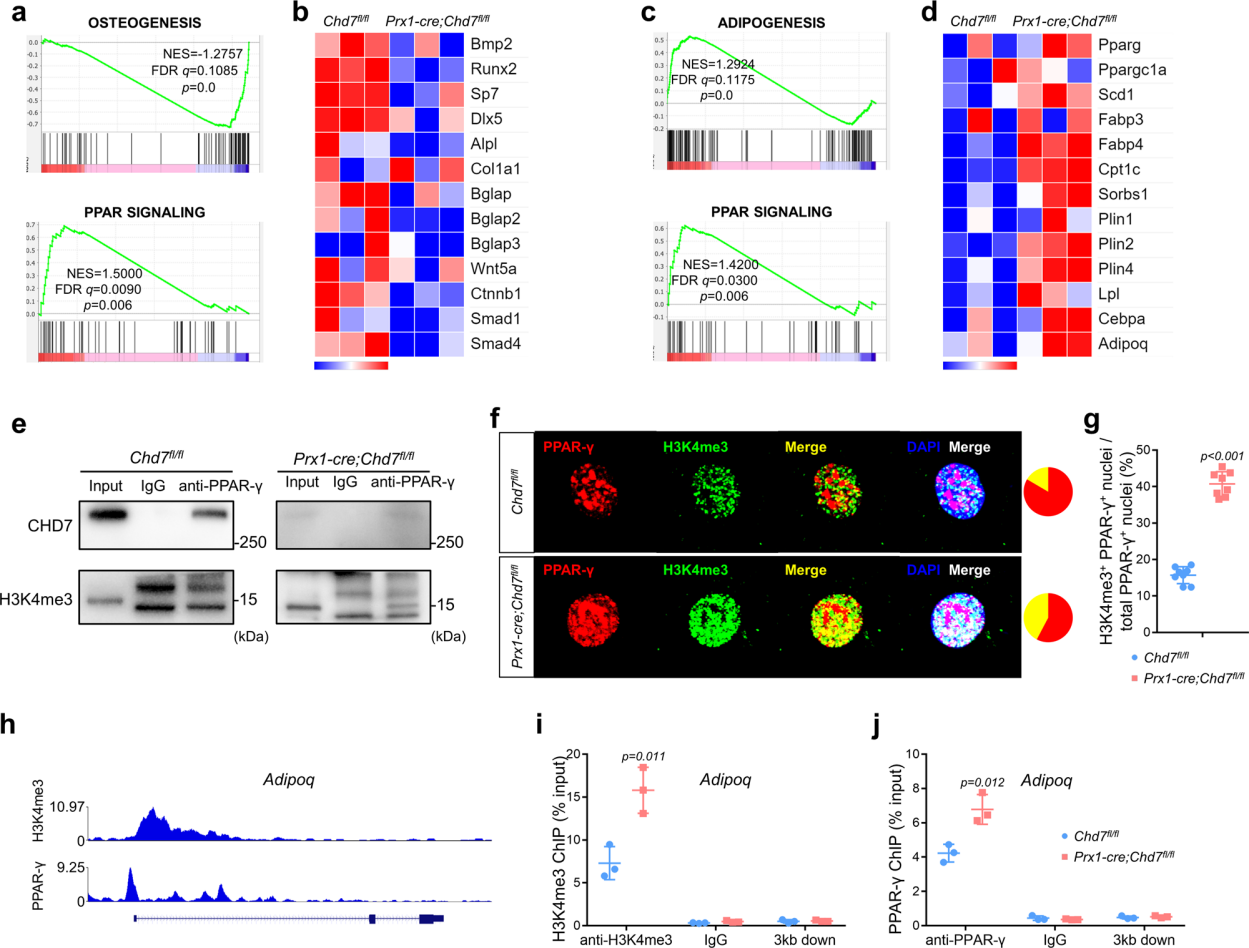
CHD7 suppresses the transactivation of PPAR-γ. **a** GSEA showing decreased enrichment of osteogenesis-related genes and increased enrichment of PPAR-regulated genes in the *Chd7*-deficient MSCs under osteogenic conditions. **b** Heatmap of representative osteogenesis-associated genes in the *Chd7*-deficient MSCs under osteogenic conditions. **c** GSEA showing increased enrichment of adipogenesis-related genes and PPAR-regulated genes in the *Chd7*-deficient MSCs under adipogenic conditions. **d** Heatmap of representative PPAR signaling pathway-associated genes in the *Chd7*-deficient MSCs under adipogenic conditions. **e** Immunoprecipitation assay in MSCs from the *Chd7*^*fl/fl*^ and *Prx1-cre;Chd7*^*fl/fl*^ mice. Cell extracts were immunoprecipitated with anti-PPAR-γ antibodies. Immunoprecipitates were detected by western blotting with the indicated antibodies. **f** Representative immunofluorescence staining of PPAR-γ and H3K4me3 in in MSCs from the *Chd7*^*fl/fl*^ and *Prx1-cre;Chd7*^*fl/fl*^ mice (Scale bar, 20 μm). **g** Quantification of (**f**), showing the proportion of H3K4me3^+^ PPAR-γ^+^ nuclei among the total PPAR-γ^+^ nuclei (*n* = 8). **h** ChIP-seq results indicating that H3K4me3 and PPAR-γ coaggregate near the promoter region of *Adipoq* by WashU EpiGenome Browser. **i** ChIP-qPCR showing that knockout of *Chd7* increases the occupancy of H3K4me3 in the promoter regions of *Adipoq* (*n* = 3). **j** ChIP-qPCR showing that knockout of *Chd7* increases the occupancy of PPAR-γ in the promoter regions of *Adipoq* (*n* = 3). Data are shown as the mean ± S.D.; *p* value by two-tailed Student’s *t* test.

Next, we performed RNA-seq of MSCs to identify the critical mechanism. Gene set enrichment analysis (GSEA) confirmed the restricted osteogenesis in *Prx1-cre;Chd7*^*fl/fl*^ MSCs after osteogenic induction (Fig. 5a, b). Interestingly, significant enhancement of the PPAR signaling pathway was found in the *Prx1-cre;Chd7*^*fl/fl*^ group, indicating that the PPAR family might play a crucial role in the formation of the abovementioned phenotype (Fig. 5a).

Meanwhile, among MSCs after adipogenic induction, significant enhancement of the PPAR signaling pathway was also found in the *Prx1-cre;Chd7*^*fl/fl*^ group, along with enhancement of the adipogenesis pathway and increased expression of downstream genes of PPAR-γ (Fig. 5c, d).

Next, we sought to verify whether CHD7 directly interacts with PPAR-γ by conducting a coimmunoprecipitation (co-IP) assay. In MSCs isolated from *Chd7*^*fl/fl*^ mice, CHD7 was successfully detected in PPAR-γ immunoprecipitates (Fig. 5e). However, H3K4me3 was barely observed in PPAR-γ immunoprecipitates, indicating that PPAR-γ might not be activated in wild-type MSCs. In contrast, H3K4me3 was detected in PPAR-γ immunoprecipitates in the *Chd7*-deficient MSCs (Fig. 5e). To intuitively observe the altered binding pattern of PPAR-γ and H3K4me3, we conducted IF staining to detect their colocalization within the nucleus. In the *Prx1-cre;Chd7*^*fl/fl*^ MSCs, the merged rates of PPAR-γ and H3K4me3 were significantly higher than those in wild-type MSCs, in accordance with the co-IP assay (Fig. 5f, g).

Next, we aimed to determine how this binding alteration regulates cell fate decisions. By analyzing ChIP-sequencing (ChIP-seq) data of *Mus musculus* (GSM2104246 and GSM1571714)^29,30^, we confirmed that H3K4me3 and PPAR-γ could coaggregate at the promotor region of *Adipoq* (Fig. 5h). On this basis, we performed a ChIP-PCR assay on the MSCs. The enrichment of H3K4me3 and PPAR-γ at the promoter regions of *Adipoq* was significantly increased in MSCs isolated from *Prx1-cre;Chd7*^*fl/fl*^ mice (Fig. 5i, j).

### PPAR-γ inhibition partially rescues the phenotype of *Chd7*-deficient MSCs

**Fig. 6:**
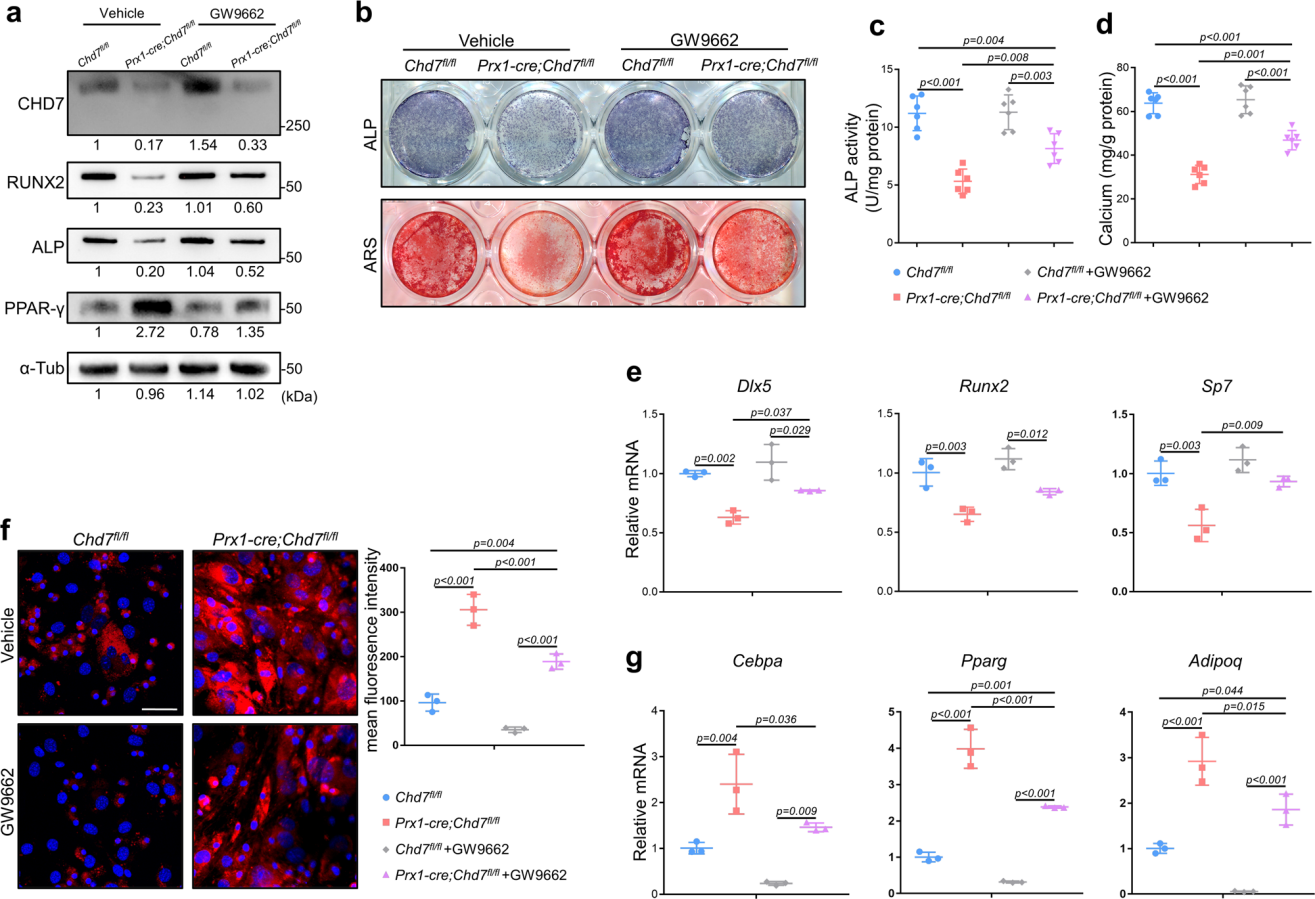
PPAR-γ inhibition partially rescues the phenotype of *Chd7*-deficient MSCs. **a** Western blot analysis and quantification of CHD7, RUNX2, alkaline phosphatase, and PPAR-γ in wild type and *Chd7*-deficient bone marrow MSCs treated with vehicle or a PPAR-γ inhibitor (GW9662). **b** Representative images of ALP and ARS staining of treated MSCs. **c** Quantitative analyses of the ALP activity in treated MSCs (*n* = 6 biologically independent MSC samples). **d** Quantitative analyses of the mineralization in treated MSCs (*n* = 6 biologically independent MSC samples). **e** qRT-PCR analyses of the mRNA expression of *Dlx5, Runx2*, and *Sp7* in treated MSCs under osteogenic conditions (*n* = 3 biologically independent MSC samples). **f** Representative images and quantitative analyses of Nile red staining in treated MSCs. Scale bar, 50 μm (*n* = 3 biologically independent MSC slides). **g** qRT-PCR analyses of the mRNA expression of *Cebpa, Pparg*, and *Adipoq* in treated MSCs under adipogenic conditions (*n* = 3 biologically independent MSC samples). Data are shown as the mean ± S.D.; *p* value by one-way ANOVA with Tukey’s post hoc tests for multiple comparisons.

To verify that activated PPAR-γ contributed to pathogenic phenotype upon *Chd7* depletion, we treated the *Prx1-cre;Chd7*^*fl/fl*^ MSCs with the PPAR-γ-specific antagonist GW9662. Although the protein level of CHD7 in the *Prx1-cre;Chd7*^*fl/fl*^ MSCs was unchanged, the expression of PPAR-γ was significantly restricted. Meanwhile, the PPAR-γ antagonist partially recovered the expression of RUNX2 and alkaline phosphatase (Fig. 6a).

After osteogenic induction, the increases in ALP, ARS staining, and ALP activity indicated the partially restored osteogenic differentiation of the treated *Chd7*-deleted MSCs (Fig. 6b–d). Meanwhile, the upregulated expression of osteogenic markers, e.g., *Dlx5, Runx2, Sp7*, strengthened the recovered osteogenic potential of treated *Chd7*-deficient MSCs (Fig. 6e).

After adipogenic induction, the weakened intensity of Nile red staining demonstrated decreased lipid droplet in the treated *Chd7*-deleted MSCs (Fig. 4f). Meanwhile, downregulated expression of classic adipogenic genes, e.g., *Cebpa, Pparg, Adipoq*, indicated the restricted adipogenic potential of the treated *Prx1-cre;Chd7*^*fl/fl*^ MSCs (Fig. 4g).

### Administration of a PPAR-γ inhibitor partially rescues the skeletal phenotype of *Prx1-cre;Chd7*^*fl/fl*^ mice

**Fig. 7:**
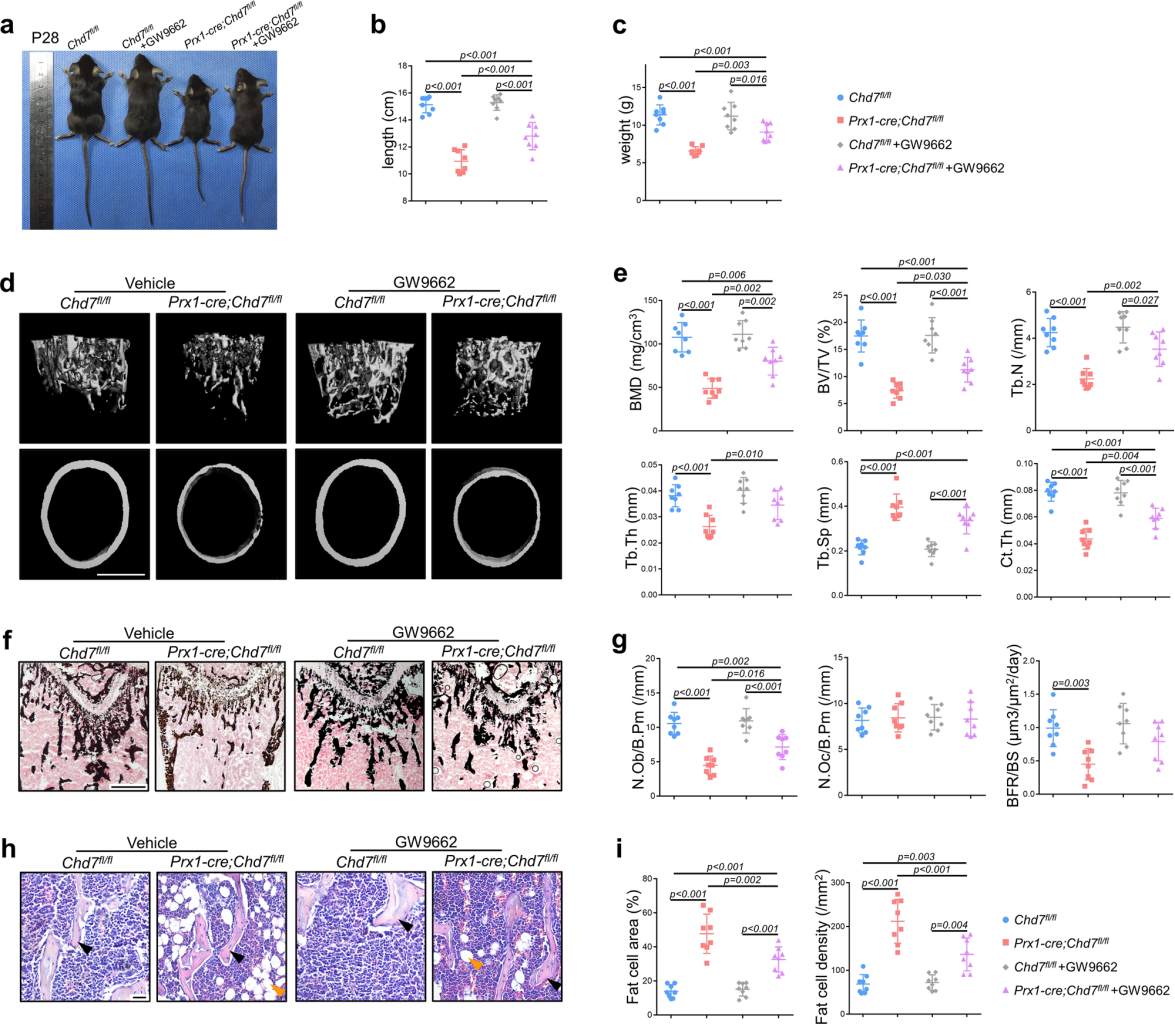
Administration of a PPAR-γ inhibitor partially rescues the skeletal phenotype of *Prx1-cre;Chd7*^*fl/fl*^ mice. **a** Representative images of the *Chd7*^*fl/fl*^ and *Prx1-cre;Chd7*^*fl/fl*^ mice at 4 weeks old, treated with vehicle or a PPAR-γ inhibitor (GW9662) (*n* = 8). **b** Length of the treated *Chd7*^*fl/fl*^ and *Prx1-cre;Chd7*^*fl/fl*^ mice at 4 weeks old (*n* = 8). **c** Weight of the treated *Chd7*^*fl/fl*^ and *Prx1-cre;Chd7*^*fl/fl*^ mice at 4 weeks old (*n* = 8). **d** Representative microCT images of distal femurs and midshaft cortical bone from the treated *Chd7*^*fl/fl*^ and *Prx1-cre;Chd7*^*fl/fl*^ mice at 4 weeks old (Scale bar, 500 μm). **e** Quantitative microCT analyses of the distal end of the femurs from the treated *Chd7*^*fl/fl*^ and *Prx1-cre;Chd7*^*fl/fl*^ mice at 4 weeks old (*n* = 8). **f** Von Kossa staining of trabecular bone from the femoral metaphysis in the treated *Chd7*^*fl/fl*^ and *Prx1-cre;Chd7*^*fl/fl*^ mice at 4 weeks old (Scale bar, 500 μm). **g** Histomorphological analysis and dynamic osteogenic index of trabecular bone from the femoral metaphysis in the treated *Chd7*^*fl/fl*^ and *Prx1-cre;Chd7*^*fl/fl*^ mice at 4 weeks old, including BFR, osteoblast, and osteoclast numbers (*n* = 8). **h** Representative images and quantitative measurements of adipocytes in the distal femur marrow in the treated *Chd7*^*fl/fl*^ and *Prx1-cre;Chd7*^*fl/fl*^ mice at 4 weeks old. Blacks arrows indicate trabecular bones, and orange arrows indicate marrow adipose tissues (Scale bar, 50 μm). **i** Quantitative data were obtained using the ImageJ software, including the number and area of adipocytes in the distal marrow per tissue area (*n* = 8). Data are shown as the mean ± S.D.; *p* value by one-way ANOVA with Tukey’s post hoc tests for multiple comparisons.

Intraperitoneal injection of the PPAR-γ inhibitor GW9662 partially recovered the body size and weight of the *Prx1-cre;Chd7*^*fl/fl*^ mice (Fig. 7a–c). MicroCT analysis of the femur metaphysis showed partially recovered trabecular and cortical bone in the treated *Prx1-cre;Chd7*^*fl/fl*^ mice, as shown by recovered skeletal parameters, e.g., BMD, BV/TV, Tb.N, Tb.Th, Ct.Th, and Tb.Sp (Fig. 7d, e).

The Von Kossa staining validated the restored bone mass of the treated *Prx1-cre;Chd7*^*fl/fl*^ mice (Fig. 7f). The treated *Prx1-cre;Chd7*^*fl/fl*^ mice also showed recovered skeletal histomorphometric features, manifested as elevated BFR and increased osteoblast numbers (Fig. 7g).

Moreover, decreased MAT accumulation was also observed in femurs from the treated *Prx1-cre;Chd7*^*fl/fl*^ mice (Fig. 7h). The number and density of bone marrow adipocytes were evidently reduced in the treated *Prx1-cre;Chd7*^*fl/fl*^ mice (Fig. 7i). Consequently, the three major manifestations of *Chd7* depletion in MSCs—whole-body skeletal system development disorder, low bone mass, and high marrow adiposity—could be partially rescued by administration of a PPAR-γ inhibitor.

## Discussion

As a dynamic process, stem cell differentiation is regulated by activating or suppressing a specific locus and dividing the genome into transcriptionally active or inactive chromadomains^31,32^. Epigenetic regulation plays an essential role in the growth and development of multiple organ systems in mammals, e.g., DNA methylation, histone modification, chromosome remodeling, and RNA interference^33,34^. As a hotspot in current research, the methylation and acetylation of histone lysines is a crucial modification at the tails of histones, playing an important role in stem cell differentiation and even cell fate decisions^35,36^. Generally, H3K4me1 is considered as a marker of enhancers, while H3K4me2 and H3K4me3 modifications are mostly enriched at promoters near transcription initiation sites to activate specific gene expression^20,37–39^. Acetylated histone H3 at lysine 27 (H3K27ac) is defined as a superenhancer that upregulates gene expression, while H3K9me3 and H3K27me3 are universally recognized as suppressive transcriptional modifications^36,40–44^.

Accumulating evidence has uncovered the regulatory role of CHD7 in epigenetics. As reported previously, in mammals, CHD7 could be recruited at the promoters or superenhancers of the transcriptionally active region of specific genes to increase or decrease transcription^20,45^. Existing studies of CHD7 preferentially shed light on nervous system development and the differentiation of neural stem cells and neural crest stem cells, and CHD7 has been proven to be crucial for neurogenesis and adult neural stem cell maintenance^9,10,13,46,47^. Widely observed in clinical work, the alteration of CHD7 expression levels in specific cell lineages leads to severe malfunction or diseases, e.g., CHARGE syndrome, Kallmann syndrome, autism spectrum disorder, developmental disorders of multiple organ systems, and tumorigenesis^13,15,45–49^. Some of these conditions were caused by the mutation of *CHD7*, while others resulted from changes in CHD7 histone modification or chromatin remodeling functions^45,48–50^.

Here, we discovered that CHD7 regulates osteogenesis and cell fate decisions in bone marrow MSCs by suppressing the PPAR-γ signaling pathway. By using different temporal markers of osteogenic stem cells, *Prx1* and *Sp7*, we established two lines of transgenic mice to conditionally knockout *Chd7* from the limb bud mesenchyme at a very early time or from preosteoblasts at the initial stage of differentiation^27,28^. We found that loss of *Chd7* in MSCs disrupted the cell fate decision in mice, leading to the pathological manifestations of osteoporosis, e.g., low bone mass with an impaired osteogenic capacity, as well as high marrow adiposity with an enhanced adipogenic ability, revealing the effective and specific regulation of CHD7 on MSCs.

Previously, PPAR-γ was reported to act as a pivotal nuclear receptor and regulate stem cell differentiation and cell fate decisions^51^. This process relies on the association with coactivators or corepressors cooperating with the methylation and acetylation of histone lysine^52^. Admittedly, osteogenesis, adipogenesis, and chondrogenesis are three major directions of the multidirectional differentiation potential of MSCs. The activation of PPAR-γ primarily drives the cell fate of MSCs to adipogenesis^52,53^. However, via the inhibiting function of PPAR-γ of specific progenitor cytokines or other corepressors, the cell fate of MSCs favoring adipogenic differentiation could also be reversed toward osteogenic differentiation^53,54^. Consistent with our findings, the enhanced PPAR signaling pathway was consistent with the phenotype of the *Prx1-cre;Chd7*^*fl/fl*^ mice in vivo and in vitro. Further, the administration of a PPAR-γ antagonist largely converted the biased MSC adipogenic differentiation and allowed osteogenesis to dominate, which also further confirmed the PPAR signaling pathway as an innovative mechanism whereby CHD7-mediated histone modification manipulated the cell lineage allocation of bone marrow MSCs.

Our findings indicated that when CHD7 bound PPAR-γ, the interaction between PPAR-γ and H3K4me3 was reduced, and consequently, the expression of the downstream adipogenic genes were downregulated, while that of osteogenic-related genes was upregulated, leading to the osteogenic differentiation of MSCs. In contrast, when CHD7 was depleted in MSCs, PPAR-γ colocalized with and contacted H3K4me3 and then activated the transcription of downstream adipogenic genes, leading to the adipogenic differentiation of MSCs (Supplementary Fig. 4a).

Notably, no significant difference (*p* > 0.05) in the osteoclast number (N.Oc/B.Pm) was found between the *Prx1-cre;Chd7*^*fl/fl*^ mice and their *Chd7*^*fl/fl*^ control littermates. We speculated that such minor alterations might not be the major factor of the low bone mass in the *Prx1-cre;Chd7*^*fl/fl*^ mice. According to a previous study, bone marrow adipocytes might be the source of RANKL^23^. Therefore, the minor alteration of the osteoclast number in the *Prx1-cre;Chd7*^*fl/fl*^ mice might be a side effect of MAT accumulation, which deserves further research.

Certain limitations in our work should be listed to provide ideas for further research. The MAT accumulation in bone marrow suggested that there might be specific influences of CHD7 on adipocytes. Conditional knockout of *Chd7* in adipose tissue in vivo would be a prospective research direction. Moreover, the lack of specimens from patients was a limitation of this research. As previously reported, CHARGE syndrome is mainly caused by the *CHD7* mutation in neural crest stem cells, while patients with *CHD7* mutations in MSCs have not been studied.

In conclusion, we demonstrate that conditional knockout of *Chd7* in bone marrow MSCs leads to the activation of the PPAR signaling pathway. Such alteration would promote the MSC differentiation balance toward adipogenesis, resulting in whole body developmental disorders of the skeletal system, low bone mass, and high marrow adipose tissue. Our research provided evidence that CHD7 regulates stem cell differentiation and cell fate decisions and revealed the functional association between histone modification and osteoporosis, which might shed light on therapeutic strategies for bone pathological disorders.

## Methods

### Generation of conditional *Chd7* knockout mice

Based on the CRISPR-Cas9 approach, *Chd7*^*fl/+*^ mice were generated with a C57BL6/J background^55–57^. Briefly, two sgRNAs were designed to target regions either upstream or downstream of exon 2 of *Chd7* with the aid of a CRISPR design tool (http://crispr.mit.edu). Then, these molecules were screened for on-target activity via a Universal CRISPR Activity Assay (UCATM, Biocytogen, Inc., Beijing). Donor vectors containing LoxP flanking exon 2 mixed with sgRNAs and Cas9 mRNA were coinjected into the cytoplasm of single-celled C57BL6/J zygotes. Such injected zygotes were transferred into ovarian ducts of Kunming pseudopregnant female mice to generate F0 mice. F0 mice with the expected genotype identified by tail genomic DNA PCR and sequencing were then mated with C57BL6/J mice to obtain germline-transmitted F1 founders. Both PCR genotyping and Southern blot examination were applied to further confirm the correct genotype of the generated *Chd7*^*fl/+*^ mice.

*Prx1-cre* and *Sp7-cre* mice were purchased from the Jackson Laboratory (Bar Harbor, ME) and were crossed with *Chd7*^*fl/+*^ mice for more than three generations to obtain *Prx1-cre;Chd7*^*fl/fl*^ and *Sp7-cre;Chd7*^*fl/fl*^ conditional knockout mice^27,28^. The genotypes were identified by PCR amplification of tail genomic DNA. Primers for the *Chd7* knockout allele and Cre transgene genotyping are listed in Supplementary Table 1. All mice were propagated and raised in specific pathogen-free facilities.

Animal experiments in this study were approved by the Institutional Review Board of West China Hospital of Stomatology, Sichuan University. All studies were approved by the Subcommittee on Research and Animal Care (SRAC) of Sichuan University. All procedures were in accordance with relevant guidelines and regulations.

### Whole-mount skeletal staining

Mouse pups were harvested at postnatal day 2. After removal of the skin and viscera, the whole mount of the pups was fixed with 95% ethanol. After being processed with acetone, the specimens were stained with Alcian blue (Solarbio) solution and decolorized with 70% and 95% ethanol. After being processed with potassium hydroxide solution, the specimens were stained with Alizarin Red S (ARS) (Solarbio) solution. After being processed with potassium hydroxide solution and glycerol, the specimens were imaged with an Epson Perfection V370 Photo Scanner. Length of humeri, femurs, and tibiae was measured with ImageJ (National Institutes of Health)^58^.

### MicroCT

Mice were sacrificed at 4 weeks old and intraperitoneally injected with calcein (10 mg/kg of body weight; Sigma) at 7 days and 2 days before sacrifice. The collected bone tissues were fixed in 4% paraformaldehyde for 24 h and then transferred to phosphate-buffered saline (PBS) at 4 °C before scanning. According to the guidelines, microCT analysis was performed via a μCT50 microCT system (Scanco Medical, Bassersdorf, Switzerland) with a spatial resolution of 8 μm (55 kV, 114 mA, 500 ms integration time). The volume of interest (VOI) of femurs was defined as a cylindrical area at the distal region of the growth plate, 1200 µm thickness for spongiosa and 200 µm thickness for cortical bone. Bone volume was calculated within the delimited VOI. Data analysis and reconstructed images were processed on IMARIS software (version 9.1.2; Oxford Instruments). Quantification of cranial defect was calculated with ImageJ (National Institutes of Health), by measuring the hole area in the cranium in the reconstructed images of skulls^3,44,59,60^.

### Undecalcified histomorphological analysis

After microCT scanning, femurs were successively dehydrated in 15% and 30% sucrose solutions and then embedded in NEG-50 (Thermo Scientific) at −80 °C. Specimens were cut into 5-µm-thick sections with a freezing microtome (CM3050 S; Leica) with a tungsten steel blade at –35 °C and were stuck to adhesive film (Cryofilm; Section-Lab Co., Ltd.).

Sections of undecalcified bone specimens were mainly processed for Von Kossa staining and analysis of double labeling. The VOI was defined as the distal end of femurs. Calcium deposits were visualized according to the Von Kossa/nuclear fast red staining method and were photographed by microscopy (BX53; Olympus). The dynamic osteogenic index was defined as the distance between 2 calcein bands by microscopy with fluorescence excitation (BX53; Olympus) and was analyzed with OsteoMeasure software (OsteoMetrics; Decatur, GA)^26,59,61^.

### Decalcified histomorphological analysis

After being fixed in 4% polyoxymethylene for 24 h and then decalcified in 10% EDTA for 2 weeks at 4 °C, femurs and tibiae were dehydrated in ethanol and xylene by Excelsior AS (Thermo Scientific). Embedded in paraffin, bone specimens were cut into 5-µm-thick sections with a paraffin microtome (RM2255; Leica)^3,5^.

Sections of decalcified bone specimens were mainly processed for hematoxylin and eosin (HE) staining, tartrate-resistant acid phosphatase (TRAP) staining, IHC staining, and IF staining. The VOI was defined as the distal end of femurs. According to the IHC and IF staining protocol, slides were incubated in sodium citrate antigen retrieval solution at 100 °C for 10 min and then incubated with rabbit anti-CHD7 antibody (Abcam, ab117522, 1:200). IF staining slides were imaged by laser scanning confocal microscopy (FV3000; Olympus), and other staining slides were photographed by microscopy (BX53; Olympus) and analyzed with OsteoMeasure software (OsteoMetrics; Decatur, GA)^5,26^.

### Cell culture and flow cytometry

Primary MSCs were isolated by flushing the bone marrow of femurs, tibiae, and humeri. The bone marrow suspension was cultured in 21 cm^2^ petri dishes in alpha minimum Eagle’s medium (α-MEM, HyClone) with 10% fetal bovine serum (FBS, Gibco), 100 units/mL penicillin, and 100 μg/mL streptomycin. After incubation at 37 °C with 5% CO_2_, the culture medium was changed every 48 h. MSCs at passage 3 were used for subsequent research^3,62,63^.

After trypsin digestion, centrifugation and resuspension, the cultured cells were equally divided into five tubes. One tube was used as blank control, and the other four tubes were added with corresponding antibodies and incubated in the dark at 37 °C for 10 min. PBS were added in each tube, and then samples were loaded into the Attune NxT Flow Cytometer (Thermo Scientific). Results were analyzed with Attune NxT Software (Thermo Scientific). The antibodies and concentrations were as follows: FITC anti-mouse/rat CD29 Antibody (BioLegend, 102205, 1:500), PE anti-mouse CD90.2 Antibody (BioLegend, 105307, 1:500), PE anti-mouse CD31 Antibody (BioLegend, 102507, 1:500), APC anti-mouse CD45 Antibody (BioLegend, 103111, 1:500).

For osteogenic induction, MSCs were cultured with osteogenic medium supplemented with 50 μM ascorbic acid (Sigma), 10 nM dexamethasone (Sigma), and 10 mM β-glycerophosphate (Sigma). For adipogenic induction, MSCs were treated with adipogenic medium containing 10 μg/ml insulin (Sigma), 1 μM dexamethasone (Sigma), and 0.5 μM isobutylmethylxanthin (Sigma)^3,26,63^.

### ALP, ARS, and Nile red staining

MSCs fixed with 4% paraformaldehyde in 24-pore plates were then stained with a BCIP/NBT Alkaline Phosphatase Color Development Kit (Beyotime) after 7 days of osteogenic induction. The reaction was terminated after 15 min of light-free incubation at room temperature, and the pore plates were imaged with an Epson Perfection V370 Photo Scanner. Quantitative analysis of ALP activity was processed with a BCA Protein Assay Kit (Beyotime) and Alkaline Phosphatase Assay Kit (Beyotime). The BCA curve was obtained from the absorbance of the BCA protein concentration gradient. The corresponding ALP activity was calculated from the standard curve of ALP absorbance by reaction with 0.5 mM p-nitrophenyl phosphate^59,62^.

MSCs in 24-pore plates were fixed with 4% paraformaldehyde and then stained with Alizarin Red S solution (Cayagen) at room temperature after 21 days of osteogenic induction. The pore plates were imaged with an Epson Perfection V370 Photo Scanner. The calcium nodules were detained by cetylpyridinium chloride for 15 minutes to quantify the calcium concentration. The absorbance at 562 nm was measured with a Multiskan Sky Microplate Spectrophotometer (Thermo Scientific), in contrast with the standard calcium absorbance curve^5,26^.

For Nile red staining, MSCs on glass coverslips were fixed with 4% paraformaldehyde and then stained with Nile red solution (Solarbio) at 37 °C for 10 min after 21 days of adipogenic induction. After DAPI staining and mounting, images were obtained with laser scanning confocal microscopy (FV3000; Olympus) and were analyzed with ImageJ (National Institutes of Health)^64^.

### Cell immunofluorescence staining

MSCs on glass coverslips were fixed with 4% paraformaldehyde and then permeabilized with 0.5% Triton X-100 in PBS at room temperature for 10 minutes. MSCs were incubated with 1% BSA, 5% goat serum, and 0.1% Triton X-100 in PBS at room temperature for a 30-minute block. MSCs were incubated with primary antibodies from different species in blocking buffer at 4 °C overnight. After removal of the primary antibodies and rinsing with PBS the next day, MSCs were successively incubated with Alexa Fluor 488 goat anti-mouse antibody (Abcam, ab150113, 1:100) and Alexa Fluor 647 goat anti-rabbit antibody (Abcam, ab150083, 1:100) in the dark at room temperature for 1 h. After DAPI staining and mounting, images were obtained with laser scanning confocal microscopy (FV3000; Olympus) and were analyzed with ImageJ (National Institutes of Health)^5^. The primary antibodies and concentrations are as follows: mouse anti-PPAR-γ antibody (Santa Cruz, sc-271392, 1:100) and rabbit anti-tri-methylhistone H3 (Lys4) antibody (CST, #9751, 1:100).

### Quantitative RT-PCR and RNA-seq

Total RNA was isolated with TRIzol Reagent (Invitrogen) 5 days after osteogenic or adipogenic induction. RNA was reverse transcribed via a PrimeScript RT reagent Kit (TaKaRa). Quantitative RT-PCR was processed with SYBR Premix Ex Taq (TaKaRa) and LightCycler 96 (Roche). The housekeeping gene *GAPDH* was used as the baseline to quantitatively analyze the gene expression of the osetogenic group, and *36B4* was used for the adipogenic group^59,62^.

For RNA sequencing, 12 RNA samples of MSCs in 4 groups were prepared according to the manufacturer’s protocol of a NEBNext Ultra RNA Library Prep Kit for Illumina (USA). RNA samples were subjected to HiSeq 2500 (Illumina). FastQC (v0.11.5) and the FASTX toolkit (0.0.13) were applied for quality control. Further analysis, e.g., KEGG enrichment, GO enrichment, heatmaps, and GSEA, was performed to explore the downstream pathways on this basis^3,26^.

### Western blot and co-IP

The total protein of MSCs was collected with a protein extraction kit (PE001, SAB Biotech) and was then heated with SDS-PAGE Sample Loading Buffer (Beyotime) at 100 °C for 5 min. After gel electrophoresis separation, protein was transferred to a PVDF membrane (Millipore) via Bio-Rad Powerpac HC. After antigen blocking, the membranes were incubated with the primary antibody described below at 4 °C overnight. After a 1-h incubation with HRP-labeled goat anti-rabbit IgG or goat anti-mouse IgG (Beyotime) at room temperature, the membranes were exposed via ChemiDoc XRS + (Bio-Rad) to detect the designated protein expression level. The primary antibodies and concentrations were as follows: rabbit anti-α-tubulin antibody (Beyotime, AF0001, 1:5000), rabbit anti-CHD7 antibody (Abcam, ab117522, 1:1000), mouse anti-RUNX2 antibody (Santa Cruz, sc-101145, 1: 1000), mouse anti-alkaline phosphatase antibody (Santa Cruz, sc-271431, 1:1000), rabbit anti-PPAR-γ antibody (CST, #2443, 1:1000), mouse anti-PPAR-γ antibody (Santa Cruz, sc-271392, 1:1000), and rabbit anti-tri-methylhistone H3 (Lys4) antibody (CST, #9751, 1:1000)^3,62^.

For the co-IP assay, MSCs were lysed in mild lysis buffer (MLB) with protease/phosphatase inhibitor cocktail (CST, #5872, 1:100) for 10 min with gentle shaking at 4 °C. After centrifugation at 14,000 × *g* for 10 min, 10% of the supernatant was heated with SDS–PAGE sample loading buffer (Beyotime) at 100 °C for 5 min as input. The remaining 90% of supernatant was collected and incubated with IgG or selected antibody overnight at 4 °C with mixing. After washes with MLB, 5 μg Pierce Protein A/G Magnetic Beads (Thermo Scientific) was added to each sample and incubated for 1 h at 4 °C with mixing. After collection and washing, the beads were heated with SDS-PAGE Sample Loading Buffer (Beyotime) at 100 °C for 5 min for subsequent western blot analysis. The co-IP antibodies were rabbit (DA1E) mAb IgG XP® Isotype Control (CST, #3900, 1:100) and rabbit anti-PPAR-γ antibody (CST, #2443, 1:100)^65^.

### ChIP assay

ChIP-seq data were obtained from GSM2104246 and GSM1571714 and were analyzed with WashU EpiGenome Browser (Washington University) to ascertain the binding site and the experimental design of ChIP-PCR^29,30^. According to the manufacturer’s protocol of the Magna ChIP HiSens Chromatin Immunoprecipitation kit (Millipore), 2 × 10^6^ cells were harvested in each ChIP reaction. Protein and DNA of each sample were crosslinked by applying 37% formaldehyde solution. SDS lysis buffer with protease/phosphatase inhibitor cocktail (CST, 1:100) was added after cell harvesting. After restriction enzyme digestion, centrifugation, and precipitation with beads, the precipitated DNA samples were quantified with specific primers via real-time PCR^5^.

### Rescue strategy in vitro and in vivo

Based on the verification of PPAR-γ signaling pathway, MSCs were treated with a PPAR-γ specific antagonist GW9662 (MedChemExpress) to rescue the phenotype in vitro. The application concentration was 10 μM, dissolved in DMSO^66^.

To partially rescue the general body phenotype in vivo, we intraperitoneally injected 3 mg/kg/day GW9662 into the female *Prx1-cre;Chd7*^*fl/fl*^ and control littermates from postnatal day 10 until 4 weeks old before sacrifice. Hereafter, microCT and decalcified and undecalcified histomorphological analyses were conducted on the specimens to clarify the rescue effect^67,68^.

### Statistical analyses

All data were calculated as the mean ± standard deviation (SD). Statistical differences were analyzed via Student’s *t* test for independent sample tests or one-way ANOVA with Tukey’s post hoc tests for multiple comparisons. A *p* value <0.05 was considered statistically significant^3,62^.

### Reporting summary

Further information on research design is available in the Nature Research Reporting Summary linked to this article.

## Supplementary information

Supplementary Information

Reporting Summary

## Data Availability

The data that support this study are available from the corresponding authors upon reasonable request. The RNA-seq data generated in this study have been deposited in the NCBI database under accession code GSE167886. The ChIP-seq data were obtained from the publicly available sequencing datasets in NCBI database under accession code GSM2104246 and GSM1571714. Source data are provided with this paper.

## Source data

Source Data
