# Supplementary material for: CHD7 regulates bone-fat balance by suppressing PPAR-γ signaling
Source: Nat Commun. 2022 Apr 13;13:1989. doi: 10.1038/s41467-022-29633-6 (PMC9007978; doi:10.1038/s41467-022-29633-6)
Supplement: Supplementary file 1 — Supplementary Information [file 41467_2022_29633_MOESM1_ESM.pdf]

Supplementary Information

of

CHD7 Regulates Bone-Fat Balance by Suppressing the PPAR-γ Signaling Pathway

Liu C et al.

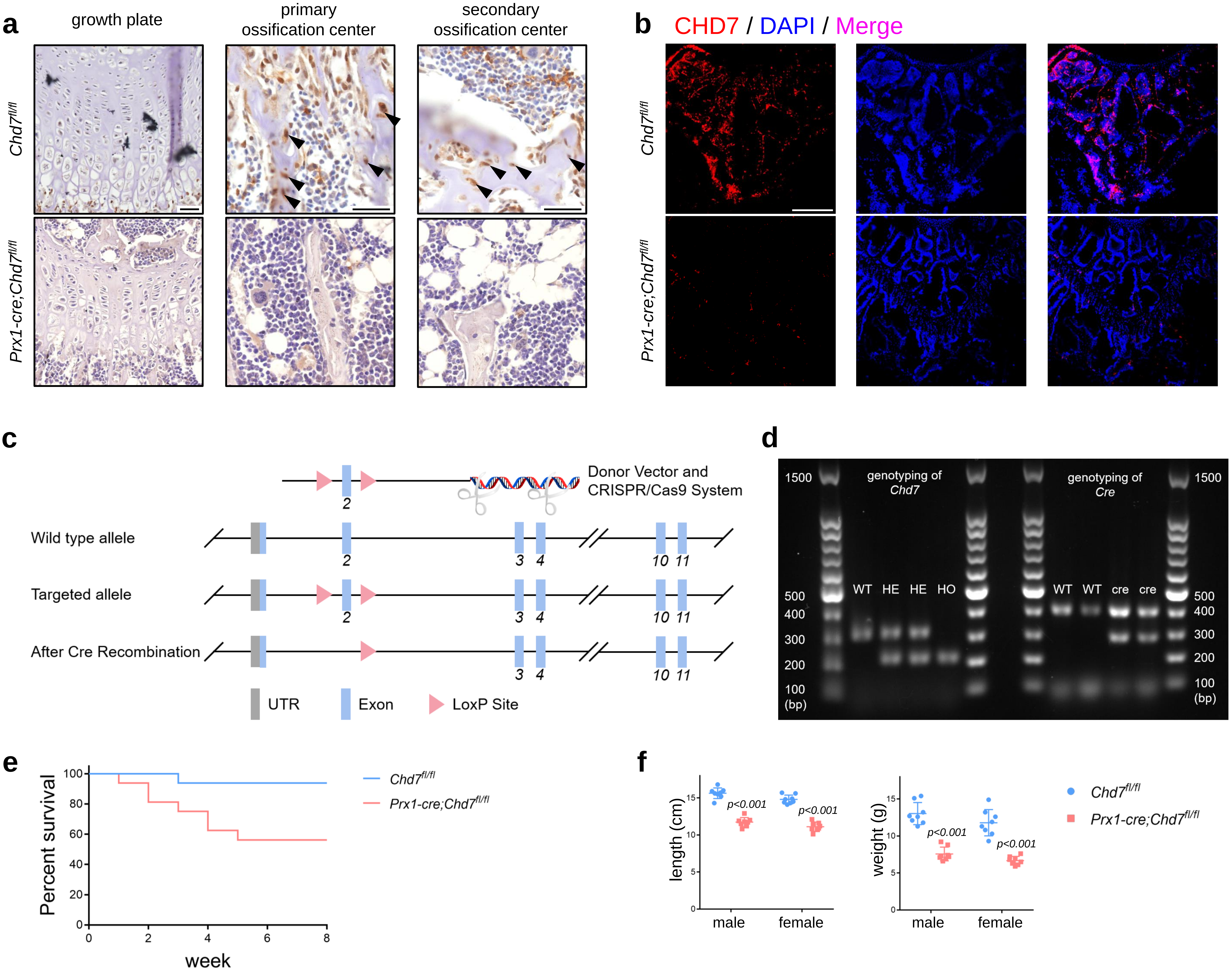

Supplementary Figure 1 Generation of conditional *Chd7* knockout mice.
a. Representative immunohistochemical staining images revealed that CHD7 was present in the bone marrow and osteoblasts of mice at 4 weeks of age but was marginally expressed in chondrocytes at the growth plate. Blacks arrows indicate CHD7^{+} osteoblasts and osteocytes (Scale bars, 50 μm) (three independent experiments). **b.** Representative immunofluorescence staining images revealed that CHD7 was present in bone marrow and osteoblasts of mice at 4 weeks of age but was marginally expressed in chondrocytes at the growth plate (Scale bar, 500 μm) (three independent experiments). **c.** Schematic representation of the conditional *Chd7* knockout strategy. Exon 2 was deleted after Cre-mediated recombination, resulting in translation termination. **d.** PCR genotyping and Southern blot examination of the *Chd7* conditional knockout mice (three independent experiments). The specific primers and PCR procedure have been provided in Supplementary Table 1. Protocols for genotyping. **e.** Survival rate of the *Chd7*$^{fl/fl}$ and *Prx1-cre;Chd7*$^{fl/fl}$ mice (n=16). **f.** Length and weight of the *Chd7*$^{fl/fl}$ and *Prx1-cre;Chd7*$^{fl/fl}$ mice at 4 weeks old (n=8). Data are shown as the mean±SD; *p* value by two-tailed Student's t test.

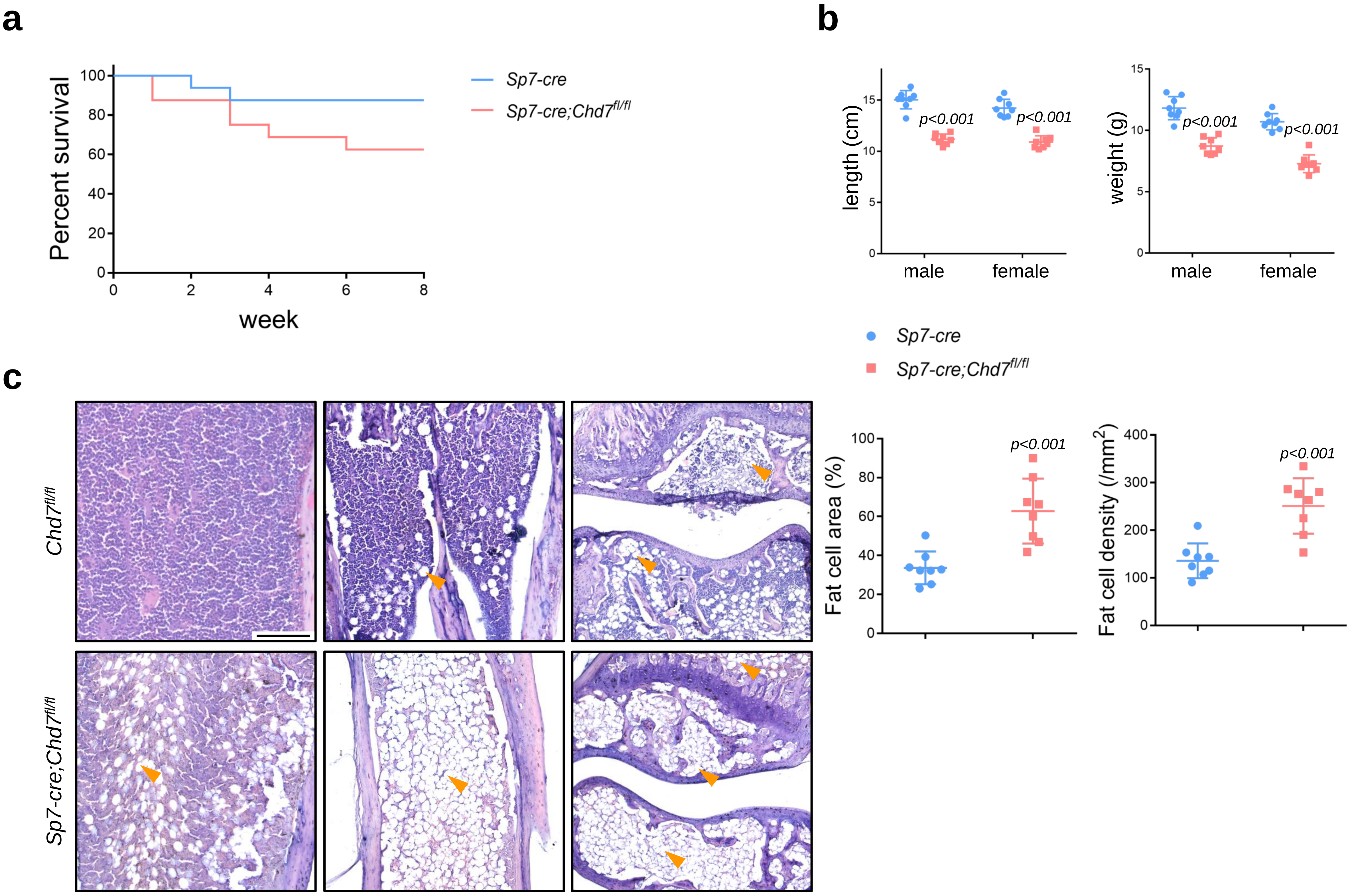

Supplementary Figure 2 Deletion of *Chd7* in pre-osteoblasts leads to smaller size and lighter weight in whole body, and severely high marrow adiposity in tibia.

a. Survival rate of the *Sp7-cre* and *Sp7-cre;Chd7*$^{fl/fl}$ mice (n=16). **b.** Length and weight of the *Sp7-cre* and *Sp7-cre;Chd7*$^{fl/fl}$ mice at 4 weeks old (n=8). **c.** Representative images and quantitative measurements of adipocytes in the mesial and distal tibia marrow in the *Sp7-cre* and *Sp7-cre;Chd7*$^{fl/fl}$ mice at 4 weeks old. Orange arrows indicate marrow adipose tissues. Quantitative data were obtained using the ImageJ software, including number and area of adipocytes in the distal marrow per tissue area (Scale bar, 200 μm) (n=8). Data are shown as the mean±SD; *p* value by two-tailed Student's t test.

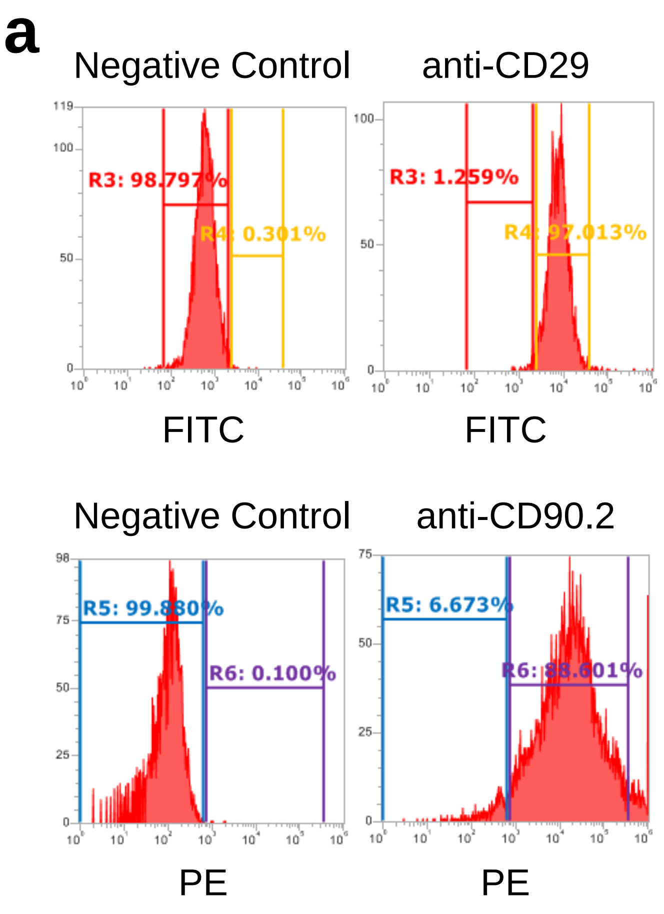

Supplementary Figure 3 Flow cytometry to identify the primary mouse bone marrow mesenchymal stem cells.
a. Flow cytometry to identify the primary mouse bone marrow mesenchymal stem cells with anti-CD29 and anti-CD90.2 antibodies.

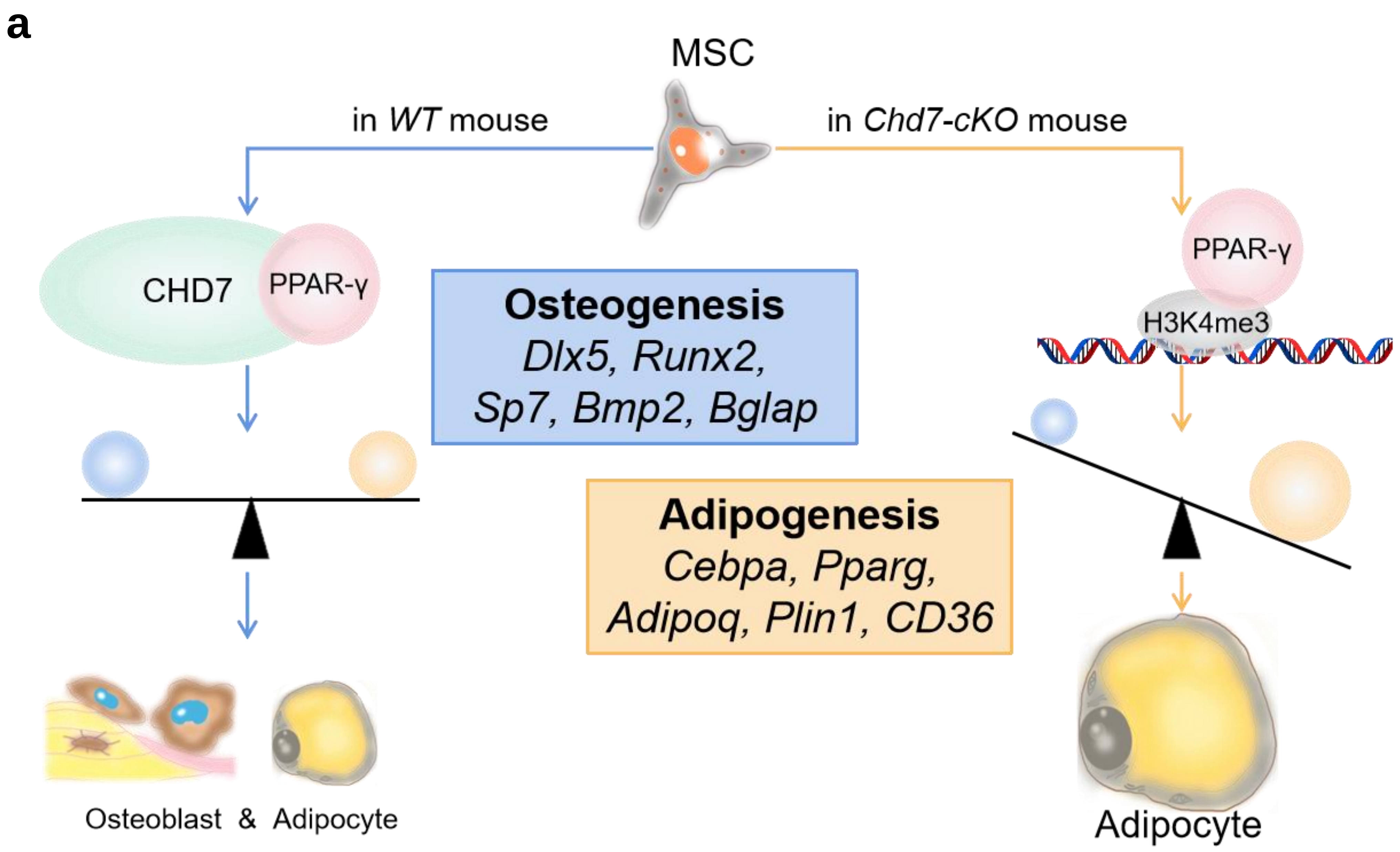

Supplementary Figure 4 CHD7 Regulates Bone-fat Balance via Suppressing PPAR-γ Signaling Pathway.
a. A mechanistic model by which CHD7 regulates osteogenesis and cell fate decisions in bone marrow mesenchymal stem cells by suppressing the PPAR-γ signaling pathway. When CHD7 bound PPAR-γ, the interaction between PPAR-γ and H3K4me3 was reduced, and consequently, the expression of the downstream adipogenic genes were downregulated, while that of osteogenic-related genes was upregulated, leading to the osteogenic differentiation of MSCs, shown as the left side of the flowchart. In contrast, when CHD7 was depleted in MSCs, PPAR-γ colocalized with and contacted H3K4me3 and then activated the transcription of downstream adipogenic genes, leading to the adipogenic differentiation of MSCs, shown as the right side of the flowchart. This process can be demonstrated by this balance scale model.

Supplementary Table 1. Protocols for genotyping

➢ Primers for genotyping of *Chd7*

PRIMER TYPE	SEQUENCE 5' → 3'
Common Forward	CAC GGC CTT GAC TTG TGA
Wild type Reverse	CAG GGG TCA CTT CCC TAA GC
Mutant Reverse	CAC CCA GGA TCC ACC TAA TAA C

➢ Genotyping of *Chd7* PCR procedure

STEP	TEMP °C	TIME	NOTE
1	94.0	2min	
2	94.0	20sec	
3	65.0	15sec	-0.5 C per cycle decrease
4	68.0	10sec	
5	--	--	repeat steps 2-4 for 10 cycles (Touchdown)
6	94.0	15sec	
7	60.0	15sec	
8	72.0	10sec	
9	--	--	repeat steps 6-8 for 28 cycles
10	72.0	2min	
11	10.0	--	hold

➢ Primers for genotyping of *Cre*

PRIMER TYPE	SEQUENCE 5' → 3'
Wild type Forward	CCA TCT GCC ACC AGC CAG
Wild type Reverse	TCG CCA TCT TCC AGC AGG
Mutant Forward	ACT GGG ATC TTC GAA CTC TTT GGA C
Mutant Reverse	GAT GTT GGG GCA CTG CTC ATT CAC C

➢ Genotyping of *Cre* PCR procedure

STEP	TEMP °C	TIME	NOTE
1	95.0	4min	
2	94.0	30sec	
3	62.0	30sec	
4	72.0	1min	
5	--	--	repeat steps 2-4 for 34 cycles
6	72.0	7min	
7	20.0	5min	
8	10.0	--	hold

Supplementary Table 2. Primers for quantitative RT-PCR

mus Gapdh-F	ACTGAGGACCAGGTTGTC
mus Gapdh-R	TGCTGTAGCCGTATTCATTG
mus Dlx5-F	CTGGCCGCTTTACAGAGAAG
mus Dlx5-R	CTGGTGACTGTGGCGAGTTA
mus Runx2-F	GGTACTTCGTCAGCATCCTATCAG
mus Runx2-R	GCTTCCGTCAGCGTCAACAC
mus Osx-F	AGAGGTTCACTCGCTCTGACGA
mus Osx-R	TTGCTCAAGTGGTCGCTTCTG
mus Bmp2-F	ACTACCAGAAACGAGTGGGAA
mus Bmp2-R	GCATCTGTTCTCGGAAAACCT
mus Bglap-F	TTGGTGCACACCTAGCAGAC
mus Bglap-R	ACCTTATTGCCCTCCTGCTT
mus 36b4-F	TGAGATTCGGGATATGCTGTTGG
mus 36b4-R	CGGGTCCTAGACCAGTGTTCT
mus Cebpa-F	ACTCCTCCTTTTCCTACCG
mus Cebpa-R	AGGAAGCAGGAATCCTCC
mus Pparg-F	CATCAGGCTTCCACTATG
mus Pparg-R	CACAGCAAGGCACTTCTG
mus Adipoq-F	CGTCACTGTTCCCAATGT
mus Adipoq-R	ACCGTGATGTGGTAAGAG
mus Plin1-F	CCTGTGGTGAGCGGGACC
mus Plin1-R	GTGGACAGCCGACGGACC
mus Cd36-F	GAG CAA CTG GTG GAT GGT TT
mus Cd36-R	GCA GAA TCA AGG GAG AGC AC
