# Supplementary material for: CHD7 regulates bone-fat balance by suppressing PPAR-γ signaling
Source: Nat Commun. 2022 Apr 13;13:1989. doi: 10.1038/s41467-022-29633-6 (PMC9007978; doi:10.1038/s41467-022-29633-6)
Supplement: Supplementary file 2 — Reporting Summary [file 41467_2022_29633_MOESM2_ESM.pdf]

nature research

Corresponding author(s): Correspondence to Ping Gong and Ning Kang. dentistgong@hotmail.com ningakang20088@163.com

Last updated by author(s): Mar 14, 2022

Reporting Summary

Nature Research wishes to improve the reproducibility of the work that we publish. This form provides structure for consistency and transparency in reporting. For further information on Nature Research policies, see our Editorial Policies and the Editorial Policy Checklist.

Statistics

For all statistical analyses, confirm that the following items are present in the figure legend, table legend, main text, or Methods section.

n/a	Confirmed	
☐	☒	The exact sample size (*n*) for each experimental group/condition, given as a discrete number and unit of measurement
☐	☒	A statement on whether measurements were taken from distinct samples or whether the same sample was measured repeatedly
☐	☒	The statistical test(s) used AND whether they are one- or two-sided<br>*Only common tests should be described solely by name; describe more complex techniques in the Methods section.*
☐	☒	A description of all covariates tested
☐	☒	A description of any assumptions or corrections, such as tests of normality and adjustment for multiple comparisons
☐	☒	A full description of the statistical parameters including central tendency (e.g. means) or other basic estimates (e.g. regression coefficient) AND variation (e.g. standard deviation) or associated estimates of uncertainty (e.g. confidence intervals)
☐	☒	For null hypothesis testing, the test statistic (e.g. *F*, *t*, *r*) with confidence intervals, effect sizes, degrees of freedom and *P* value noted<br>*Give P values as exact values whenever suitable.*
☒	☐	For Bayesian analysis, information on the choice of priors and Markov chain Monte Carlo settings
☒	☐	For hierarchical and complex designs, identification of the appropriate level for tests and full reporting of outcomes
☐	☒	Estimates of effect sizes (e.g. Cohen's *d*, Pearson's *r*), indicating how they were calculated

Our web collection on statistics for biologists contains articles on many of the points above.

Software and code

Policy information about availability of computer code

Data collection	OsteoMeasure (Ver3.1.0.2), ImageJ (v.1.6.0), HISAT2 (v.2.0.4), Ballgown software (v.3.4.0), GSEA(v.4.0.2), Attune NxT Software (Thermo Scientific)
Data analysis	GraphPad Prism; version 8.00

For manuscripts utilizing custom algorithms or software that are central to the research but not yet described in published literature, software must be made available to editors and reviewers. We strongly encourage code deposition in a community repository (e.g. GitHub). See the Nature Research guidelines for submitting code & software for further information.

Data

Policy information about availability of data

All manuscripts must include a data availability statement. This statement should provide the following information, where applicable:
- Accession codes, unique identifiers, or web links for publicly available datasets
- A list of figures that have associated raw data
- A description of any restrictions on data availability

Source data are provided with this paper.
The RNA-seq data generated in this study have been deposited in the NCBI database under accession code GSE167886 [https://www.ncbi.nlm.nih.gov/geo/query/acc.cgi?acc=GSE167886].
The ChIP-seq data were obtained from the publicly available sequencing datasets in NCBI database under accession code GSM2104246 [https://www.ncbi.nlm.nih.gov/geo/query/acc.cgi?acc=GSM2104246] and GSM1571714 [https://www.ncbi.nlm.nih.gov/geo/query/acc.cgi?acc=GSM1571714].

Field-specific reporting

Please select the one below that is the best fit for your research. If you are not sure, read the appropriate sections before making your selection.

- [x] Life sciences
- [ ] Behavioural & social sciences
- [ ] Ecological, evolutionary & environmental sciences

For a reference copy of the document with all sections, see nature.com/documents/nr-reporting-summary-flat.pdf

Life sciences study design

All studies must disclose on these points even when the disclosure is negative.

Sample size	No statistical method was used to predetermine the sample size. Sample size was determined according to previously published paper in relevant field and prior experience in our laboratory(Weiqing Liu et al,Nature Communications, 2016; Yunshu Wu et al, Nature Communications, 2018).
Data exclusions	All inclusion/exclusion criteria were pre-established and no samples or animals were excluded from the analysis.
Replication	All experiments were performed as technical or biological replicates as appropriate for the experimental design.
Randomization	Animals were allocated into control or experimental group according to their genotype. The conditional knockout mice were set as experimental group, and their wild type littermates set as control group.
Blinding	As the whole body type of the conditional knockout mice and the their wild type littermates were different from birth, were not able to carry out experiment with blinding during the raising, administration, and sacrificing period. Therefore, blinding was performed later in the outcome assessment by other investigators.

Reporting for specific materials, systems and methods

We require information from authors about some types of materials, experimental systems and methods used in many studies. Here, indicate whether each material, system or method listed is relevant to your study. If you are not sure if a list item applies to your research, read the appropriate section before selecting a response.

Materials & experimental systems

n/a	Involved in the study	
[ ]	[x]	Antibodies
[x]	[ ]	Eukaryotic cell lines
[x]	[ ]	Palaeontology and archaeology
[ ]	[x]	Animals and other organisms
[x]	[ ]	Human research participants
[x]	[ ]	Clinical data
[x]	[ ]	Dual use research of concern

Methods

n/a	Involved in the study	
[x]	[ ]	ChIP-seq
[ ]	[x]	Flow cytometry
[x]	[ ]	MRI-based neuroimaging

Antibodies

Antibodies used

For IHC or IF Staining:
rabbit anti-CHD7 antibody (abcam, ab117522, 1:200);
mouse anti-PPAR-γ antibody (Santa Cruz, sc-271392, 1:100);
rabbit anti-Tri-Methyl-Histone H3 (Lys4) antibody (CST, #9751, 1:100);

Secondary antibody: goat anti-mouse antibody Alexa Fluor 488 (abcam, ab150113, 1:100) and goat anti-rabbit antibody Alexa Fluor 647 (abcam, ab150083, 1:100);

For Co-IP or ChIP:
protease/phosphatase inhibitor cocktail (CST, #5872, 1:100);
rabbit (DA1E) mAb IgG XP® Isotype Control (CST, #3900, 1:100);
rabbit anti-PPAR-γ antibody (CST, #2443, 1: 100);

For WB:
rabbit anti-α-tubulin antibody (Beyotime, AF0001, 1:5000);
rabbit anti-CHD7 antibody (abcam, ab117522, 1: 1000);
mouse anti-RUNX2 antibody (Santa Cruz, sc-101145, 1: 1000);
mouse anti-Alkaline Phosphatase antibody (Santa Cruz, sc-271431, 1: 1000);
rabbit anti-PPAR-γ antibody (CST, #2443, 1: 1000);

	mouse anti-PPAR-γ antibody (Santa Cruz, sc-271392, 1: 1000);<br>rabbit anti-Tri-Methyl-Histone H3 (Lys4) antibody (CST, #9751, 1: 1000);<br><br>Secondary antibody: HRP-labeled Goat Anti-Rabbit IgG (Beyotime, A0208, 1:5000); HRP-labeled Goat Anti-Mouse IgG (Beyotime, A0216, 1:5000)<br><br>For flow cytometry:<br>FITC anti-mouse/rat CD29 Antibody (BioLegend, 102205; 1:500);<br>PE anti-mouse CD90.2 Antibody (BioLegend, 105307; 1:500);<br>PE anti-mouse CD31 Antibody (BioLegend, 102507; 1:500);<br>APC anti-mouse CD45 Antibody (BioLegend, 103111; 1:500)
Validation	rabbit anti-CHD7 antibody (abcam, ab117522, 1: 200); Application: ICC/IF, WB, IHC-P; Species Reactivity: Mouse, Human;<br>mouse anti-PPAR-γ antibody (Santa Cruz, sc-271392, 1: 100); Application: WB, IP, IF, ELISA; Species Reactivity: mouse, rat, human;<br>rabbit anti-Tri-Methyl-Histone H3 (Lys4) antibody (CST, #9751, 1: 100); Application: WB IHC, IF, F, ChIP, C&R; Species Reactivity: mouse, rat, human, monkey, Dm, Sc;<br>rabbit anti-PPAR-γ antibody (CST, #2443, 1: 100); Application: WB, IP, IF, ChIP; Species Reactivity: Mouse, Human;<br>rabbit anti-α-tubulin antibody (Beyotime, AF0001, 1:5000); Application: WB, ICC, IHC, IF; Species Reactivity: mouse, rat, human;<br>mouse anti-RUNX2 antibody (Santa Cruz, sc-101145, 1: 1000); WB, IP, IF, IHC-P, ELISA; Species Reactivity: mouse, rat, human;<br>mouse anti-Alkaline Phosphatase antibody (Santa Cruz, sc-271431, 1: 1000); Application: WB, IP, IF, IHC-P, ELISA; Species Reactivity: mouse, rat, human;

Animals and other organisms

Policy information about studies involving animals; ARRIVE guidelines recommended for reporting animal research

Laboratory animals	Based on CRISPR-Cas9 approach, Chd7fl/+ mice were generated with C57BL6/J background. Prx1-Cre and Sp7-cre mice were purchased from the Jackson Laboratory (Bar Harbor, ME) and were crossed with Chd7fl/+ mice for more than three generations to obtain Prx1-Cre;Chd7fl/fl mice and Sp7-Cre;Chd7fl/fl mice conditional knockout mice. Female and male mice (P0-P28) were used in our study.Temperature (23t2"C) and humidity (55%) were held constant in animal housing.
Wild animals	This study did not involve wild animals.
Field-collected samples	This study did not involve samples collected from the field.
Ethics oversight	Approvals for all the protocols were obtained from the Subcommittee on Research and Animal Care (SRAC) of Sichuan University.

Note that full information on the approval of the study protocol must also be provided in the manuscript.

Flow Cytometry

Plots

Confirm that:

- [x] The axis labels state the marker and fluorochrome used (e.g. CD4-FITC).
- [x] The axis scales are clearly visible. Include numbers along axes only for bottom left plot of group (a 'group' is an analysis of identical markers).
- [ ] All plots are contour plots with outliers or pseudocolor plots.
- [x] A numerical value for number of cells or percentage (with statistics) is provided.

Methodology

Sample preparation	After trypsin digestion, centrifugation and resuspension, the cultured cells were equally divided into five tubes. One tube was used as blank control, and the other four tubes was added with corresponding antibodies and incubated in the dark at 37 °C for 10 minutes. PBS were added in each tube before loading samples.
Instrument	Attune NxT Flow Cytometer (Thermo Scientific)
Software	Attune NxT Software (Thermo Scientific)
Cell population abundance	Around 6×10^4 cells were loaded in each sample.
Gating strategy	Gate was determined by excluding more than 99.5% cells in the blank control sample. Cells whose relative fluorescence intensity is above the gate were defined to be positive.

- [x] Tick this box to confirm that a figure exemplifying the gating strategy is provided in the Supplementary Information.
